# Ion-pair complexes of oxidorhenate(v) with phenolate–oxazoline ligands: synthesis, solvatochromism and catalytic properties

**DOI:** 10.1039/d6dt02275h

**Published:** 2026-10-27

**Authors:** Tobias A. Doliner, Anna M. Schlegl, Kristin Kogler, Milan Milovanović, A. D. Boese, Ferdinand Belaj, Antoine Dupé, Peter E. Hartmann, Jörg A. Schachner

**Affiliations:** a Institute of Chemistry, University of Graz Schubertstraße 1 8010 Graz Austria joerg.schachner@uni-graz.at; b Innovation Center of the Faculty of Chemistry, Belgrade Studentski trg 12-16 11158 Belgrade Serbia

## Abstract

Ion-pair complexes, in which an organic cation exists entirely in the second coordination sphere of an anionic metal complex, represent a structurally and electronically distinct compound class, whose solution behavior is influenced by solvent polarity and hydrogen-bonding interactions. Their capacity to undergo ion pair charge transfer (IPCT) transitions makes them attractive model complexes for ion-pairing equilibria and potential active species in catalytic cycles. Herein we report the synthesis, structural characterization, spectroscopic investigation and catalytic evaluation of the oxidorhenate(v) outer-sphere ion pair complexes (H_2_L1)[ReOCl_3_(L1)] (1) and (H_2_L2)[ReOCl_3_(L2)] (2). Both complexes crystallize as contact ion pairs stabilized by N–H⋯Cl and O–H⋯Cl hydrogen bonds. The hydrogen-bond-accepting Re–Cl bonds are significantly elongated as shown by single crystal X-ray diffraction analysis, indicating enhanced lability at these sites. Both complexes display a striking solvatochromism, assigned by UV/Vis spectroscopy to an IPCT transition operative exclusively in apolar media (*λ*_max_ = 555 nm, *ε* = 2700 L mol^−1^ cm^−1^ for 1; *λ*_max_ = 577 nm, *ε* = 920 L mol^−1^ cm^−1^ for 2, solvent: CHCl_3_). Additionally, DFT calculations across a series of solvents establish the thermodynamic basis for the observed solvent-dependent ion-pair equilibria. Cyclic voltammetry reveals quasi-reversible Re(v/vi) couples for **1** in both solvents and for **2** in MeCN (*E*_1/2_ = +0.431 V for 1; +0.258 V for 2 in MeCN *vs.* Fc^+^/Fc). Both complexes catalyze perchlorate reduction with dimethyl sulfide, achieving 92% conversion (TOF 0.37 h^−1^) for 1 and 55% (TOF 0.22 h^−1^) for 2. ^1^H NMR monitoring shows that 1 is not converted into the bis-chelate complex during catalysis.

## Abstract 2

Ion pairing in oxidorhenium(v) complexes triggers striking solvatochromism *via* ion-pair charge transfer (IPCT), a phenomenon mechanistically elucidated through combined experiment and theory.

## Graphical abstract

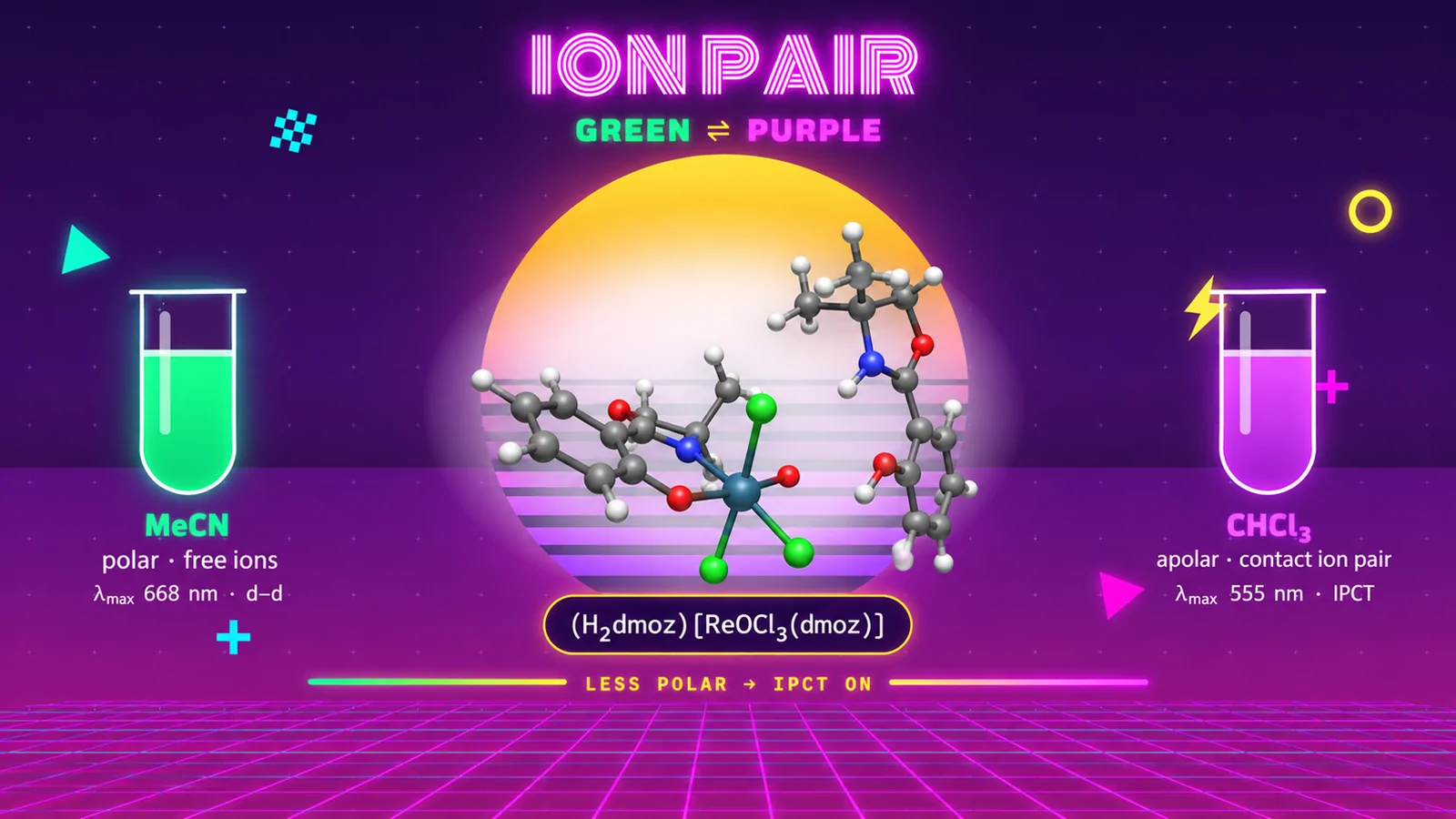

## Introduction

Oxidorhenium(v) complexes bearing bidentate phenolate–oxazoline (ON) ligands have emerged as a versatile and widely studied system for homogeneous oxygen atom transfer (OAT) catalysis, most prominently in the reduction of environmentally relevant oxyanions such as perchlorate and nitrate.^1–6^ Since the group of Abu-Omar first demonstrated the quantitative reduction of perchlorate to chloride using bis-ligated [ReOCl(L1a)_2_] (HL1a = Hoz = 2-(2′-hydroxyphenyl)-2-oxazoline, Fig. 1) and dimethyl sulfide (DMS) as the sacrificial oxygen acceptor,^1^ extensive structure–activity studies have established that both the stereoisomeric form and the electronic character of the bidentate ligand direct catalytic efficiency.^2–6^

**Fig. 1:**
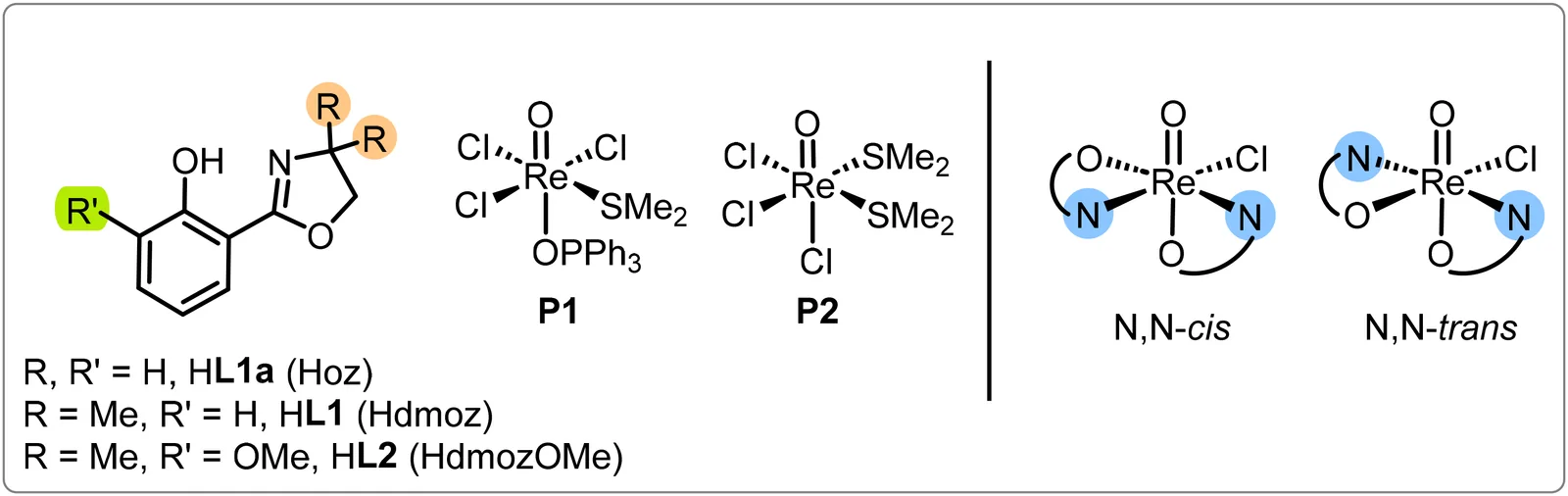
Left: structure of ligand HL1a (Hoz) pioneered by the Abu-Omar group and ligands HL1/HL2 investigated within this project; structures of commonly used precursor complexes P1/P2. Right: general depiction of the N,N-*cis* and N,N-*trans* stereoisomers of the [ReOX(ON)_2_] type.

The high activity of this compound class stems from the ability of rhenium to cycle reversibly between the Re(v) and Re(vii) oxidation states, mirroring the Mo(iv)/Mo(vi) redox couple found in biological molybdoenzymes.^7^ The structural versatility of phenolate–oxazoline chelates allows systematic tuning of both the electronic properties of the metal center and the steric environment of the coordination sphere.^3,4^ Bis-ligated N,N-*trans* isomers of the general type [ReOCl(ON)_2_], in which the two oxazoline nitrogen donors are mutually *trans* (Fig. 1), are consistently the most active perchlorate-reduction catalysts. The group of Schachner showed that the stronger *trans* influence of the phenolate oxygen labilizes the chlorido co-ligand and thereby facilitates substrate coordination.^2,5^ The 4,4-dimethyl substitution of the oxazoline ring in ligand HL1 (Hdmoz = 2-(4,4-dimethyl-4,5-dihydro-1,3-oxazol-2-yl)phenol, Fig. 1) provides the steric preference required to reliably select the N,N-*trans* isomer stereoselectively.^5,6^

In the current mechanistic understanding, the formation of bis-ligated complexes of the type [ReOCl(ON)_2_] proceeds stepwise (Scheme 1). In the first step, one equivalent of (HON) coordinates with the phenolate oxygen *trans* to the oxido ligand, generating a monoligated anionic oxidorhenate(v) intermediate of the type [ReOCl_3_(ON)]^−^. The proton released during phenolate coordination is abstracted by the second equivalent of (HON), which thereby acts as a Brønsted base rather than a nucleophile, resulting in the ion pair (H_2_ON)[ReOCl_3_(ON)] with the protonated organic cation (H_2_ON)^+^.^8,9^ In the second step, the cation coordinates to rhenium under loss of HCl, forming the bis-ligated complex [ReOCl(ON)_2_]. Liu *et al.*^10^ reported the isolation of (H_2_L1a)[ReOCl_3_(L1a)] in ethanol and Schachner *et al.*^5^ identified the analogous (H_2_ON)[ReO_4_] species. The occurrence of this intermediate directly explains the observed stereoselectivity to N,N-*trans* in the synthesis of [ReOCl(ON)_2_] complexes like [ReOCl(L1)_2_] (3). The mono-ligated anion requires a fixed oxido–phenolate *trans* arrangement that is preserved before the second ligand moiety does coordinate. Under optimized conditions like short reaction times, coordinating solvents or the use of the phosphine-free precursor [ReOCl_3_(SMe_2_)_2_] (P2), these intermediates can be isolated and characterized rather than converting further to the bis-ligated product.^2,10,11^

**Scheme 1:**
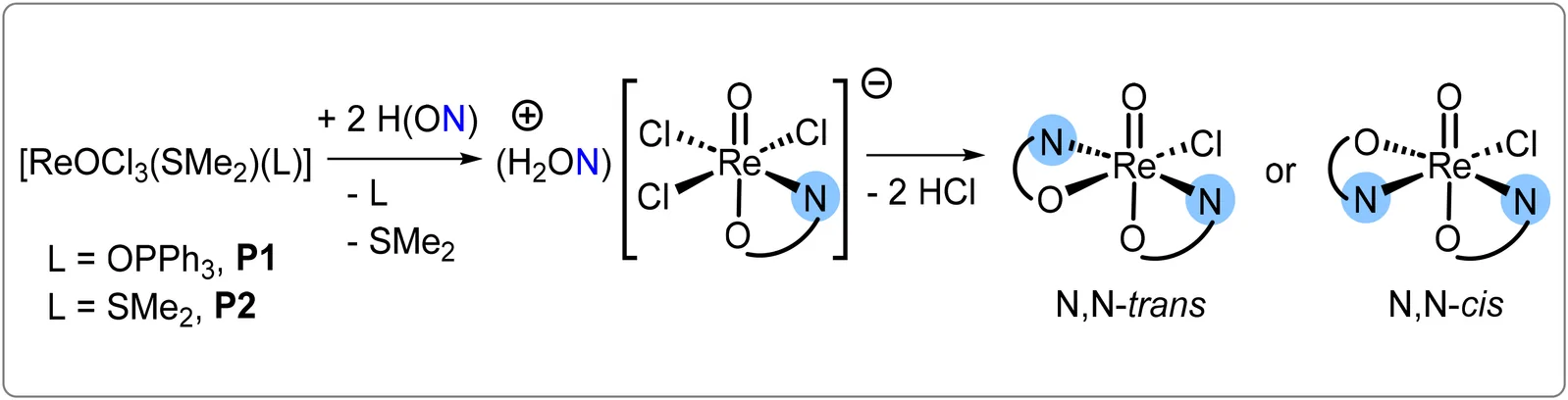
Mechanism of stepwise formation of bis-ligated complexes *via* an oxidorhenate(v) intermediate.

Such (H_2_ON)[ReOCl_3_(ON)] complexes constitute a structurally and electronically distinct class of Re(v) complexes. Notably, the protonated H_2_ON^+^ cation is not incorporated into the inner coordination sphere of rhenium, but is attached to the anionic [ReOCl_3_(ON)]^−^ complex through a network of directional N–H⋯Cl and O–H⋯Cl hydrogen bonds, yielding a so-called outer-sphere ion pair (OSIP) complex.^12,13^ Their chemical and spectroscopic properties are sensitive to solvent polarity. In media of low dielectric constant, the ions condense into intimate contact ion pairs (CIPs) stabilized by short inter-ionic distances and directional hydrogen bonds, whereas in polar solvents the ions are separated by intercalating solvent shells.^12^ A particularly attractive consequence of CIP formation is the possible occurrence of ion pair charge transfer (IPCT) transitions, in which an electron is formally transferred from a donor orbital localized on one ion to an acceptor orbital on its counter-ion.^14–16^ As such transitions are symmetry-allowed and driven by coulombic attraction between the ions, they typically give rise to intense, broad visible absorption bands with molar extinction coefficients *ε* of the order of 10^2^–10^3^ L mol^−1^ cm^−1^, which is two to three orders of magnitude larger than those of metal-centered d–d transitions. The IPCT band energy and intensity are directly linked to the donor–acceptor distance and the electronic coupling matrix element *H*_DA_, making IPCT complexes sensitive probes of ion-pairing equilibria in solution.^15,16^

The orbital basis of IPCT transitions has been characterized in detail by several benchmark systems. In the octacyanometalate(iv) complex (H_2_bpy)_2_[M(CN)_8_]·4H_2_O (M = Mo, W; H_2_bpy = 2,2′-bipyridinium), an IPCT band is observed whose energy shifts bathochromically from 501 nm in solution to ∼587 nm in the solid state as the inter-ionic donor–acceptor distance contracts upon crystallisation.^15^ The distance-dependence of the IPCT band energy has been quantified through a series of [W(CN)_8_]^2−^/bipyridinium ion pairs in which *λ*_max_ tracks the inter-ionic separation.^16^ In these systems the donor is a filled metal d-orbital of the [M(CN)_8_]^4−^ anion and the acceptor is the π* system of the bipyridinium dication; the transition can accordingly be described as a formal metal-to-ligand charge transfer across the ion pair interface.^14^ A conceptually related picture applies to the present (H_2_ON)[ReOCl_3_(ON)] complexes: here, the primary donor orbital is the Re(v) d_*xy*_ orbital (the HOMO of the anionic fragment, modified by π-bonding with the oxido and phenolate donors), while the acceptor is a ligand-centred π* orbital delocalised over the aromatic ring and the protonated iminium fragment of the H_2_ON^+^ cation.^2,17^ The IPCT excitation therefore corresponds formally to Re(v) → Re(vi) oxidation coupled to partial reduction of the organic counter-cation. This formal Re(vi) state refers only to the IPCT excited state and is not involved in the catalytic cycle, which proceeds *via* the Re(v) and Re(vii) oxidation states. Orbital overlap between these two frontier orbitals is mediated by the N–H⋯Cl and O–H⋯Cl hydrogen bonds that hold the CIP in a well-defined geometry.^17^

IPCT phenomena have been documented across a broad range of ion-pair systems, from organic donor–acceptor salts^14,18^ to polycyanometalate/bipyridinium assemblies.^15,16^ In the context of Re(v) ion pair chemistry, the complex (H_2_L1)[ReOCl_3_(L1)] (1) is particularly noteworthy: its solid-state colour (green) transforms into vivid purple upon dissolution in solvents of low polarity such as chloroform or ethyl acetate, a solvatochromism that had remained only tentatively attributed to IPCT. The methoxy-substituted analogue (H_2_L2)[ReOCl_3_(L2)] (2), in which an electron-donating –OMe group is appended to the phenolate ring, provides a direct handle for examining electronic substituent effects on the IPCT band parameters.

Herein we report the discovery and comprehensive characterization of oxidorhenate(v) outer-sphere ion pair complexes, (H_2_L1)[ReOCl_3_(L1)] (1) and (H_2_L2)[ReOCl_3_(L2)] (2), whose solution chemistry is governed by solvent-dependent ion pair equilibria. Single-crystal X-ray structures reveal contact ion pairs stabilized by a network of N–H⋯Cl and O–H⋯Cl hydrogen bonds that induce striking elongation of hydrogen-bond-accepting Re–Cl bonds (up to 0.055 Å), pre-activating the complex toward catalytic chloride dissociation. A comprehensive solvent-dependent UV/Vis investigation establishes that the vivid purple coloration of these complexes in apolar media originates from ion-pair charge transfer (IPCT) transitions with extinction coefficients (*ε* ∼10^2^ to 10^3^ L mol^−1^ cm^−1^) that dominate over conventional d–d absorption. DFT calculations across a broad range of solvents reveal a pronounced solvent-polarity dependence of CIP and bis-chelate stability. These trends reproduce the experimentally observed solvent dependence of the UV/Vis spectra and further explain the selective isolation of the CIP from polar solvents. Cyclic voltammetry demonstrates that the π-donor effect of the *ortho*-methoxy substituent on the phenolate ligand tunes the Re(v/vi) oxidation potential by 173 mV and switches the post-oxidative pathway between irreversible EC behavior and quasi-reversible electron transfer. In perchlorate reduction, both complexes act as catalysts, with **1** not being converted into the bis-chelate complex during catalysis and complex **2** showing a reduced turnover frequency (TOF 0.22 *vs.* 0.37 h^−1^).

## Results and discussion

### Synthesis of 1 and 2

Complex 1 was obtained by reacting the phosphine-free precursor [ReOCl_3_(SMe_2_)_2_] (P2) with two equivalents of Hdmoz (HL1) in dry acetonitrile at 60 °C for 1 h under N_2_, followed by stirring at room temperature for 2 h (Scheme 2). The use of P2 represents a significant practical improvement over the previously employed precursor [ReOCl_3_(OPPh_3_)(SMe_2_)] (P1), as it avoids the need to separate triphenylphosphine oxide as a by-product. Acetonitrile distinctively directs the reaction towards 1, while the use of less polar reaction solvents (CHCl_3_, toluene, CH_2_Cl_2_) resulted in intractable product mixtures. Conducting the reaction in a sealed Schlenk flask under N_2_ retards HCl loss, which would otherwise drive the second ligand coordination and result in conversion of 1 to 3. Work-up exploits the differential solubility of 1 and the side products in ethyl acetate. The light green solid was isolated in 42% yield; recrystallisation from concentrated methanol afforded crystals suitable for X-ray diffraction.

**Scheme 2:**
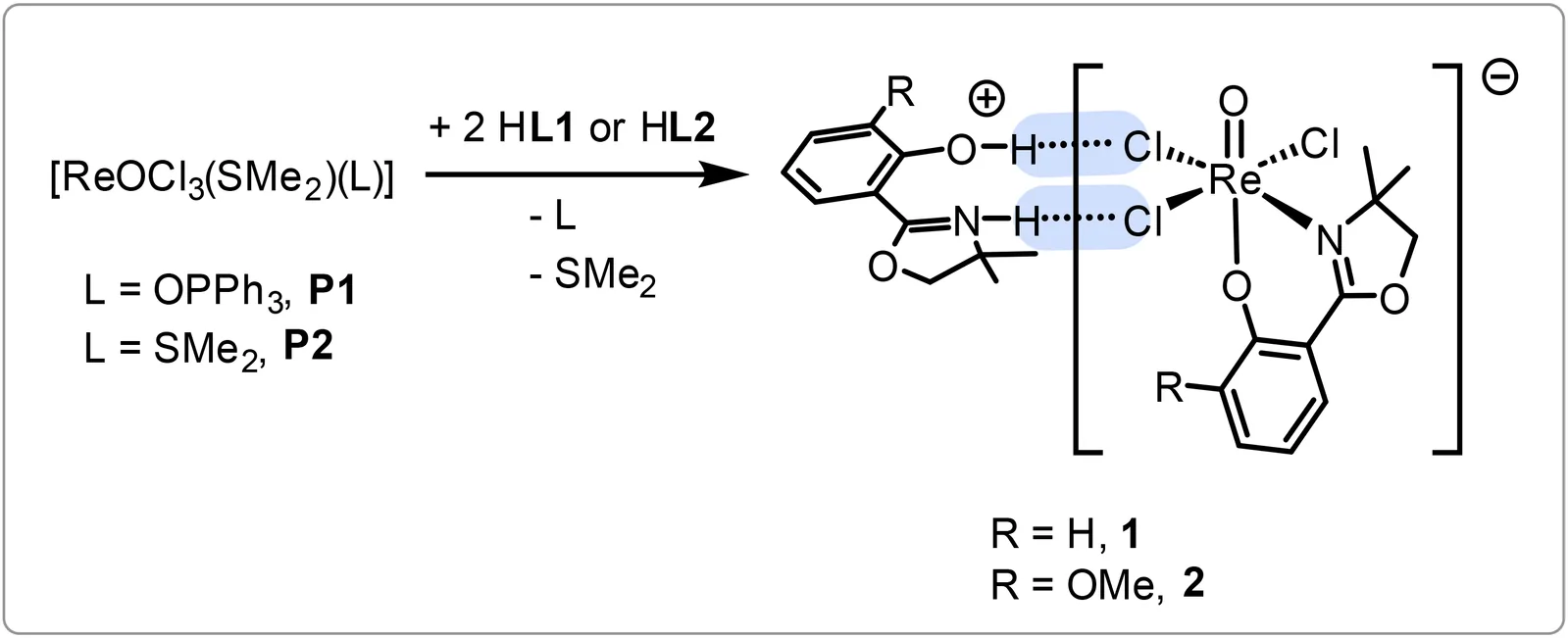
Synthesis of complexes 1 and 2.

The methoxy-substituted analogue 2 was prepared in 39% yield from [ReOCl_3_(OPPh_3_)(SMe_2_)] (P1) and 2.2 equivalents of ligand HL2 in refluxing acetone for 5 h (Scheme 2). Both acetonitrile and acetone were tested as reaction solvents for the syntheses of 1 and 2 and gave the same results. This agrees with the very similar preference for the CIP over the bis-chelate predicted by DFT for both solvents (Table 3). A notable preparative observation is that the recrystallisation solvent controls which product is isolated. Acetone affords 2, whereas recrystallisation from ethanol/isopropanol cleanly yields the neutral bis-ligated complex [ReOCl(L2)_2_] (4).

### Crystal structures of 1 and 2

Complex 1 crystallizes in the monoclinic space group *P*2_1_/*c* (*Z* = 4; *R*_1_ = 0.0256, w*R*_2_ = 0.0524, Fig. 2). The rhenium center adopts a distorted octahedral geometry. The terminal oxido ligand (*d*(Re–O1) = 1.6826(11) Å) is oriented *trans* to the phenolate oxygen (*d*(Re–O21) = 1.9651(11) Å; ∠O1–Re–O21 = 172.90(5)°), consistent with the dominant *trans* influence of terminal oxido groups in Re(v) chemistry.^5,11^ The oxazoline nitrogen (*d*(Re–N13) = 2.1215(14) Å) is *trans* to Cl2 (∠N13–Re–Cl2 = 173.47(4)°), while Cl1 and Cl3 occupy the *trans* positions (∠Cl1–Re–Cl3 = 170.18(1)°).

**Fig. 2:**
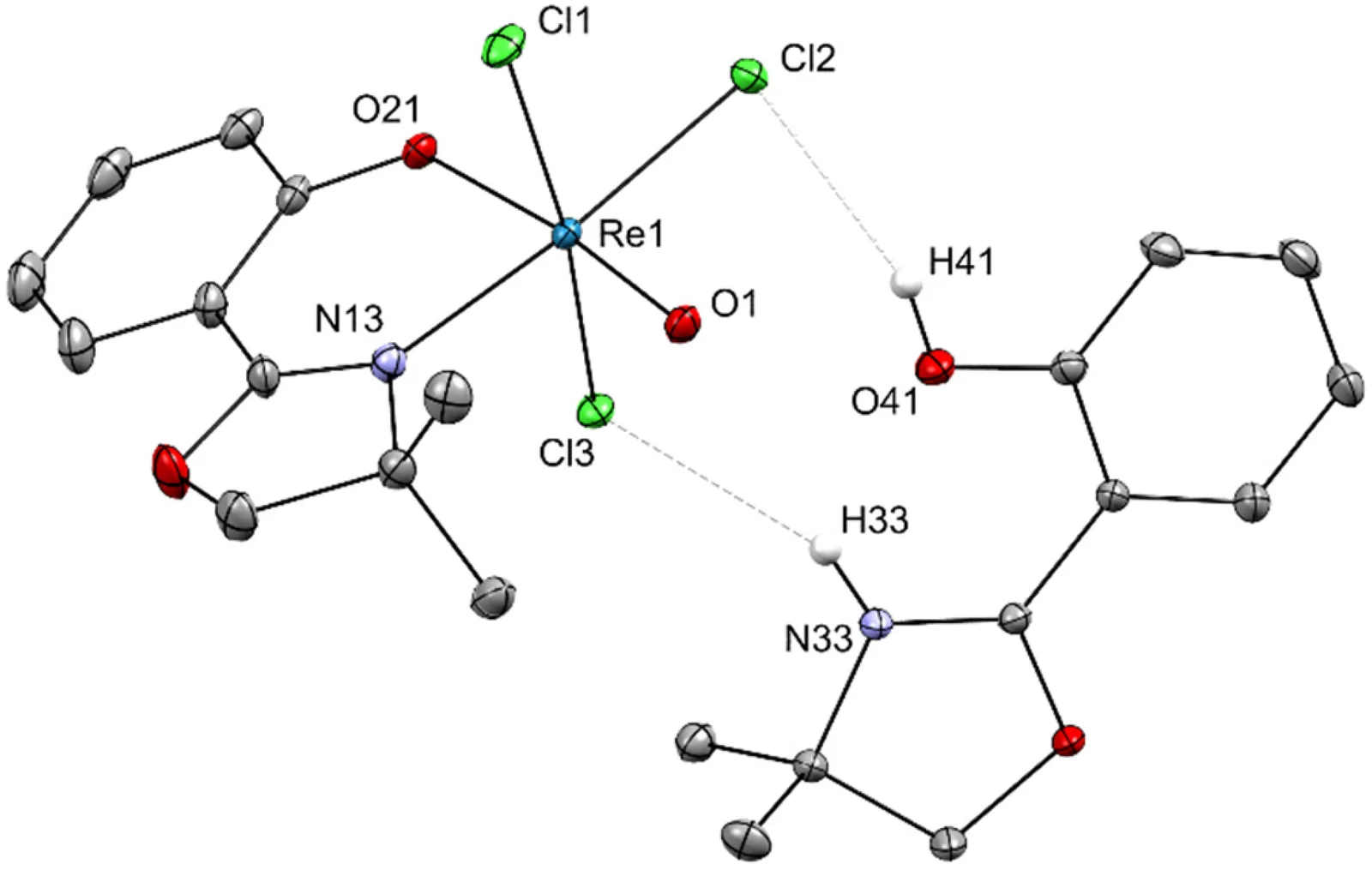
ORTEP plot of complex 1 (50% probability ellipsoids). H atoms except H33 (N–H) and H41 (O–H) are omitted; hydrogen bonding shown as dashed lines.

A structurally diagnostic feature is the pronounced spread in Re–Cl bond lengths. Re–Cl1 (2.3714(4) Å), which bears no hydrogen-bond interaction, is significantly shorter than Re–Cl2 (2.4028(4) Å) and Re–Cl3 (2.4268(4) Å), both of which accept contacts from the H_2_dmoz^+^ counter-ion: O41⋯Cl2 = 3.1720(12) Å, ∠153(3)° (O–H⋯Cl); N33⋯Cl3 = 3.2414(13) Å, ∠147(2)° (N–H⋯Cl). Since Cl2 is located *trans* to the oxazoline nitrogen atom N13, whereas Cl1 and Cl3 are mutually *trans*, only the latter two can be compared directly. Along the Cl1–Re–Cl3 axis, the hydrogen-bonded Cl3 is elongated by 0.055 Å relative to Cl1. Judged by the donor–acceptor distances, the O–H⋯Cl2 contact is in fact slightly shorter than the N–H⋯Cl3 contact, but no non-hydrogen-bonded reference in the position *trans* to N13 is available to quantify the elongation of Cl2. The elongation of the hydrogen-bond-accepting Re–Cl bonds signals enhanced lability at these sites, pre-activating the complex for catalytic chloride dissociation.^5,10^ The H_2_dmoz^+^ cation is additionally stabilized by a bifurcated N33–H33 hydrogen bond: besides the N33–H33⋯Cl3 contact, a secondary intramolecular N33–H33⋯O41 interaction (*d*(H⋯O) = 2.130 Å, ∠N–H⋯O = 119.9°) connects the protonated oxazoline nitrogen to the phenol oxygen of the same cation, forming a six-membered pseudo-ring and rigidifying the counter-cation conformation.

Complex 2 crystallizes in the chiral orthorhombic space group *P*2_1_2_1_2_1_ (*Z* = 4, *R*_1_ = 0.0286, w*R*_2_ = 0.0574, Fig. 3). The coordination geometry is again distorted octahedral, with the oxido ligand (*d*(Re–O1) = 1.684(3) Å) nearly collinear with the phenolate oxygen (*d*(Re–O21) = 1.973(3) Å; ∠O1–Re–O21 = 175.80(14)°) and the oxazoline nitrogen (*d*(Re–N13) = 2.133(4) Å) *trans* to Cl2 (∠N13–Re–Cl2 = 168.74(11)°). In contrast to 1, the three Re–Cl distances in 2 are more uniform (2.3899(11), 2.3918(12) and 2.4048(11) Å; total spread: 0.015 Å *vs.* 0.055 Å in 1). Selected bond parameters are compiled in Table 1. The MeO-H_2_dmoz^+^ counter-ion forms two directional hydrogen bonds with the anionic complex: O41⋯Cl2 = 3.070(4) Å, ∠O–H⋯Cl = 151(6)°; N33⋯Cl1 = 3.407(4) Å, ∠N–H⋯Cl = 151(4)°. Along the Cl1–Re–Cl3 axis, the hydrogen-bonded Cl1 is only 0.013 Å longer than the non-bonded Cl3, compared with 0.055 Å in 1. This small elongation agrees with the much weaker N–H⋯Cl contact in 2 (N33⋯Cl1 3.407(4) Å *vs.* N33⋯Cl3 3.2414(13) Å in 1). The MeO-H_2_dmoz^+^ cation is additionally stabilised by a bifurcated N33–H33 hydrogen bond: besides the N33–H33⋯Cl1 contact, a secondary intramolecular N33–H33⋯O41 interaction (*d*(H⋯O) = 2.05(5) Å, ∠N–H⋯O = 122(4)°) connects the protonated oxazoline nitrogen to the phenol oxygen of the same cation, forming a six-membered pseudo-ring and rigidifying the counter-cation conformation. In addition, the phenolic OH group has a short contact to the adjacent methoxy oxygen atom (O⋯O 2.61 Å), which competes with the cation–anion hydrogen bonding.

**Fig. 3:**
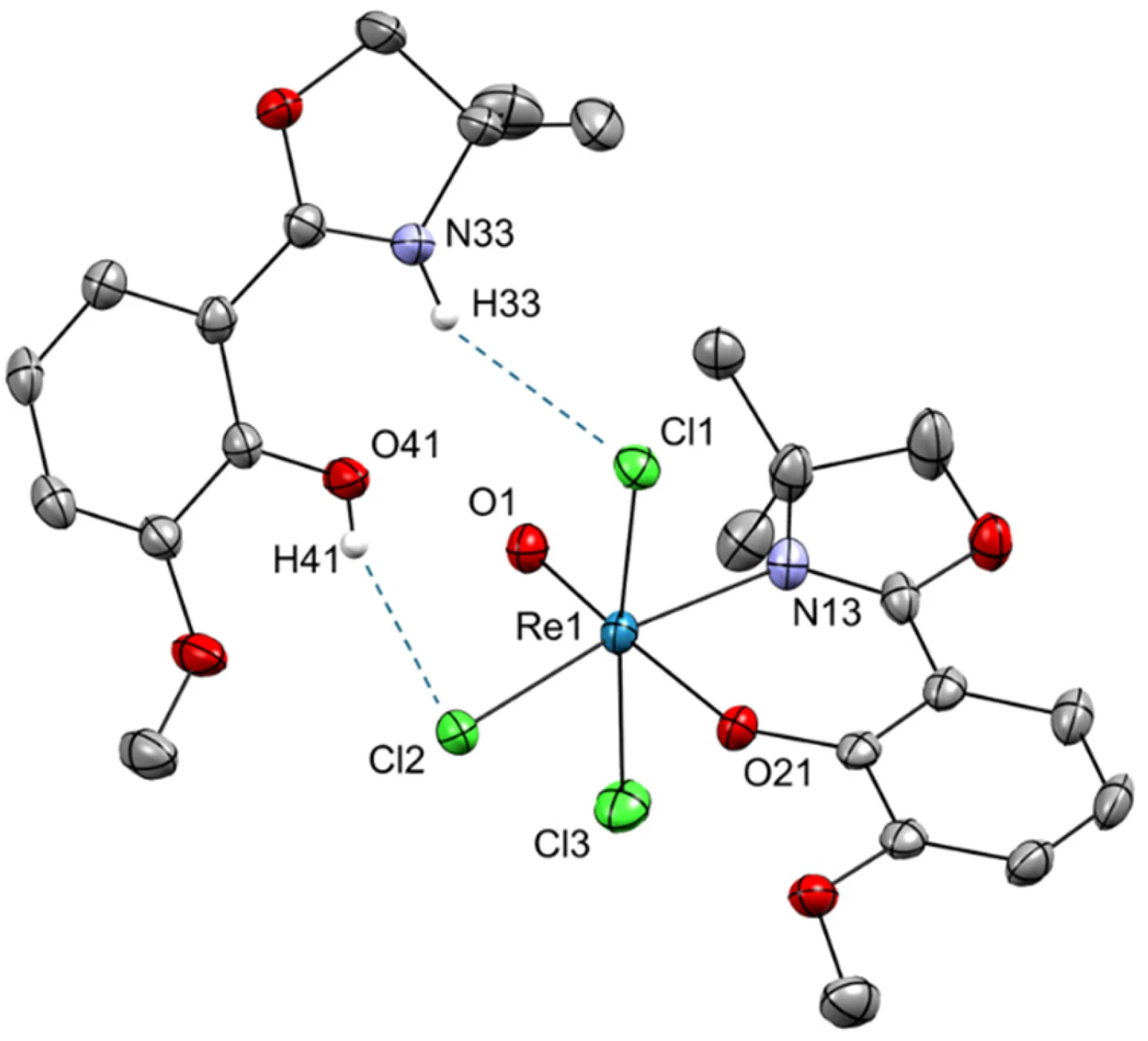
ORTEP plot of complex 2 (50% probability ellipsoids). H atoms except H-bond donors are omitted; hydrogen bonding shown as dashed lines.

**Table 1:** Selected bond lengths (Å) and angles (°) for complexes 1 and 2

Parameter	Complex 1	Complex 2
Bond lengths		
Re=O (oxido)/Å	1.6826(11)	1.684(3)
Re–O (phenolate)/Å	1.9651(11)	1.973(3)
Re–N (oxazoline)/Å	2.1215(14)	2.133(4)
Re–Cl (short)/Å	2.3714(4)	2.3899(11)
Re–Cl (mid)/Å	2.4028(4)	2.3918(12)
Re–Cl (long)/Å	2.4268(4)	2.4048(11)
Bond angles		
∠(O=Re–O)/°	172.90(5)	175.80(14)
∠(N–Re–Cl)/°	173.47(4)	168.74(11)
∠(Cl–Re–Cl)/°	170.18(1)	173.57(5)

### Solvatochromism and UV/Vis spectroscopy

Both 1 and 2 display an intense visually evident solvatochromism. The complexes are light green in the solid state and in polar solvents (MeCN, MeOH, EtOH) but turn purple in solvents with low-to-moderate dielectric constant (CHCl_3_, EtOAc, CH_2_Cl_2_). UV/Vis spectroscopy across a polarity series (Table 2 and Fig. S1, S2) gives evidence that this solvatochromism originates from IPCT transitions.

**Table 2:** UV/Vis absorption data for complexes 1 and 2

	Complex 1		Complex 2	
Solvent	*λ*_max_/nm	*ε*/L mol^−1^ cm^−1^	*λ*_max_/nm	*ε*/L mol^−1^ cm^−1^
MeCN	668	49	705	57
CHCl_3_	555	2700	577	920
EtOAc	531	1100	572	680

In MeCN (*ε*_r_ = 37.5), both complexes display a weak, time-independent absorption (*λ*_max_ = 668 nm, *ε* = 49 L mol^−1^ cm^−1^ for 1; 705 nm, *ε* = 57 L mol^−1^ cm^−1^ for 2), characteristic of the Laporte-forbidden d–d transition of the effectively free [ReOCl_3_(ON)]^−^ anion. The spectral time-independence confirms complete dissociation of the CIP in this polar medium.

In chloroform (*ε*_r_ = 4.8), an intense IPCT band develops at *λ*_max_ = 555 nm (*ε* = 2700 L mol^−1^ cm^−1^) for 1 and at 577 nm (*ε* = 920 L mol^−1^ cm^−1^) for 2. These values exceed the d–d band in acetonitrile by more than two orders of magnitude, clearly identifying a fully allowed charge-transfer transition. The band grows continuously over 11 h after dissolution, reflecting kinetically controlled assembly of the hydrogen-bonded CIP geometry required for IPCT. The subsequent decline in absorbance (beyond 15 h) is accompanied by ^1^H NMR evidence for conversion of (H_2_L1)[ReOCl_3_(L1)] (1) to [ReOCl(L1)_2_] (3). A long-term measurement of 2 in CHCl_3_ shows the same qualitative behaviour on a shorter timescale. The IPCT band at 575 nm reaches its maximum after about 2 h and decreases to about 10% of this value within 12 h (Fig. S4, SI).

In ethyl acetate (*ε*_r_ = 6.02), both 1 and 2 likewise develop a purple IPCT band, but with a distinct temporal evolution in which *λ*_max_ shifts bathochromically from 510 to 531 nm (1) and from 539 to 572 nm (2) concurrent with the absorbance increase, in contrast to chloroform where *λ*_max_ remains fixed. This suggests that the equilibrium CIP geometry is approached gradually in ethyl acetate, likely because the hydrogen-bond-accepting carbonyl group initially competes with direct ion-pair contact. The final extinction coefficients of *ε* = 1100 and 680 L mol^−1^ cm^−1^ for 1 and 2, respectively, are intermediate between the chloroform and acetonitrile values, consistent with less complete CIP formation in this moderately polar solvent.

Comparison of 1 and 2 reveals two systematic substituent effects. First, *λ*_max_ is consistently red-shifted in 2 by 22–41 nm across all solvents, reflecting a raised Re(v) d-manifold energy from the electron-donating methoxy group, which strengthens the π-donor character of the phenolate chelate. Second, *ε* is substantially lower in 2 (920 *vs.* 2700 L mol^−1^ cm^−1^ in CHCl_3_), suggesting reduced donor–acceptor electronic coupling *H*_DA_ in the methoxy complex. In Marcus–Hush theory for charge transfer, the intensity scales as *H*_DA_^2^, which depends sensitively on the spatial overlap of the donor Re d_*xy*_ orbital and the π* acceptor orbital of H_2_ON^+^. The extended π-system of the methoxyphenolate may partly delocalise the Re d-electron density, reducing the effective orbital overlap across the ion pair interface.^19^ In addition, the solid-state structures reveal a considerably weaker cation–anion hydrogen bonding in 2 (N33⋯Cl1 3.407(4) Å *vs.* N33⋯Cl3 3.2414(13) Å in 1), indicating a looser ion pair with a larger donor–acceptor distance. The intramolecular contact between the phenolic OH group and the methoxy oxygen atom in the cation of 2 competes with the cation–anion hydrogen bonds and is expected to shift the equilibrium away from the contact ion pair. Since the reported *ε* values are apparent values based on the total rhenium concentration, a smaller fraction of contact ion pairs further lowers *ε* for 2.

### Computational studies

To obtain insight into the underlying factors governing our experimental observations, we assessed the thermodynamics of the various discussed species across a series of solvents by means of density functional theory (DFT) calculations (Table 3). The considered solvents comprise four apolar ones (toluene, chloroform, dichloromethane, ethyl acetate) and four polar ones (acetone, ethanol, methanol and acetonitrile), exhibiting an overall range of relative permittivities from *ε*_r_ = 2.38 (toluene) to *ε*_r_ = 37.5 (acetonitrile). The Gibbs free energies discussed below were obtained at both 25 °C and 1.0 bar at the ωB97X-D3/dhf-TZVPP//PBE-D3BJ/dhf-SVP level of theory employing the COSMO solvation model using TURBOMOLE 7.9 (for details, see SI).^20–27^ We note that evaluating the Gibbs free energies at 60 °C or changing to a different density functional approximation (B3LYP) does not alter the qualitative picture (see SI).^28–30^

Solvent dependence of the computed Gibbs free energies of association (Δ*G*°, kJ mol^−1^) for contact ion-pair complexes 1 and 2 and bis-chelate isomers 3 and 4 relative to the fully separated ions, and relative stabilization of the CIP over the bis-chelate isomer (Δ*G*1*vs.*3; Δ*G*2*vs.*4). Values obtained at the ωB97X-D3/dhf-TZVPP//PBE-D3BJ/dhf-SVP level with the COSMO solvation model (25 °C, 1 bar)

**Table:** 

Solvent	*ε*_r_	Δ*G* CIP (1) [kJ mol^−1^]	Δ*G* bis-chelate (3) [kJ mol^−1^]	Δ*G* (1) *vs.* (3) [kJ mol^−1^]
Acetonitrile	37.5	**+11.1**	**+33.5**	−22.4
Methanol	32.6	**+9.1**	**+31.1**	−22.0
Ethanol	24.3	**+4.2**	**+25.1**	−21.0
Acetone	20.7	**+0.5**	**+21.0**	−20.5
Dichloromethane	8.93	−29.3	−13.9	−15.4
Ethyl acetate	6.02	−53.7	−42.0	−11.7
Chloroform	4.81	−71.3	−62.0	−9.2
Toluene	2.38	−152.0	−152.2	**+0.1**

**Table:** 

Solvent	*ε*_r_	Δ*G* CIP (2) [kJ mol^−1^]	Δ*G* bis-chelate (4) [kJ mol^−1^]	Δ*G* (2) *vs.* (4) [kJ mol^−1^]
Acetonitrile	37.5	**+8.2**	**+34.8**	−26.6
Methanol	32.6	**+5.6**	**+32.5**	−26.9
Ethanol	24.3	**+1.6**	**+24.3**	−25.0
Acetone	20.7	−1.3	**+22.8**	−24.1
Dichloromethane	8.93	−28.7	−10.8	−18.0
Ethyl acetate	6.02	−52.9	−37.6	−15.3
Chloroform	4.81	−67.3	−56.6	−10.6
Toluene	2.38	−132.3	−143.4	**+0.8**

Our calculations reveal a strong solvent polarity dependence of the Gibbs free energy of CIP formation from the separated ions. While for the apolar media formation of the CIP 1 is strongly exergonic (Δ*G* = −152.0 to −29.3 kJ mol^−1^), for the polar media it becomes endergonic (Δ*G* = +0.5 to +11.1 kJ mol^−1^). This energetic trend is also reflected in the hydrogen bond lengths in the optimized geometries (see SI). The same qualitative trend is encountered for methoxy-substituted CIP 2, demonstrating that ion pair stability transcends the subtle electronic differences between L1 and L2. Thus, in apolar solvents the CIP constitutes a stable arrangement, whereas in polar solvents it is predicted to dissociate into the individual ions. These findings coincide with the experimental UV/Vis studies displaying CIP formation in chloroform and ethyl acetate as opposed to CIP dissociation in acetonitrile.

A similar polarity dependence was obtained for the Gibbs energy of formation of bis-chelate 3 from the separated ions, being exergonic for apolar media (Δ*G* = −152.2 to −13.9 kJ mol^−1^) as opposed to endergonic in polar media (Δ*G* = +21.0 to +33.5 kJ mol^−1^). Moreover, for the polar solvents, CIP 1 is found to be thermodynamically significantly more stable than the bis-chelate 3 (Δ*G* = −22.4 kJ mol^−1^ for acetonitrile, the most polar considered medium). This difference in stability diminishes with solvent polarity, until the stabilities of CIP 1 and bis-chelate 3 become almost equal (Δ*G* = +0.1 kJ mol^−1^ for toluene, the most apolar considered medium). Again, the same qualitative trends are also retrieved for the methoxy-substituted species, indicating only a minor influence of the introduced functionality (see Fig. 4 and Table 3).

**Fig. 4:**
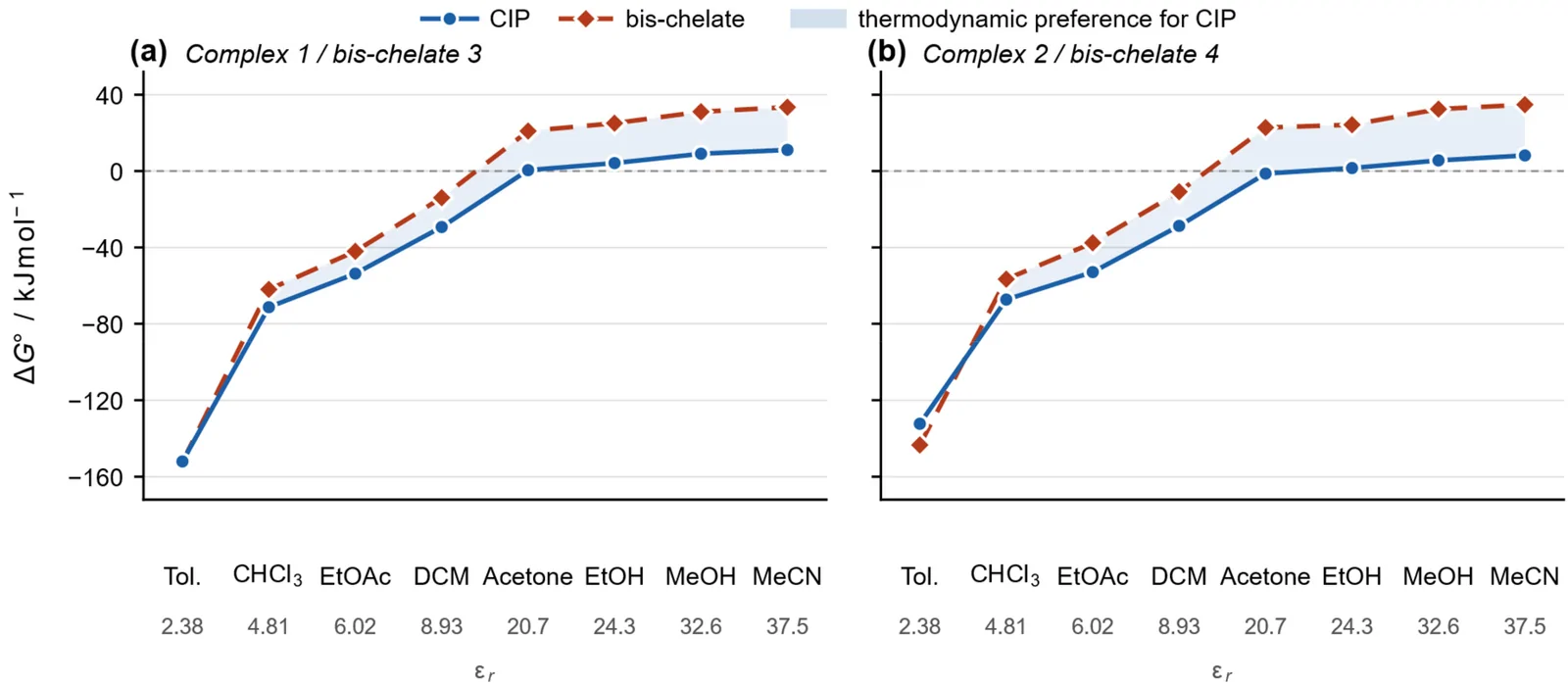
Calculated Gibbs free energies (Δ*G°*) for contact ion pair (CIP, solid line) and bis-chelate complex (dashed line) formation from separated ions as a function of relative permittivity *ε*_r_ (left to right). The shaded area denotes the thermodynamic preference of the CIP over the bis-chelate. Level of theory: ωB97X-D3/dhf-TZVPP//PBE-D3BJ/dhf-SVP, COSMO solvation model, 25 °C, 1 bar. All values in kJ mol^−1^.

Together, these findings provide a rationale for the selective CIP formation in the solvents (acetonitrile, acetone) employed in the optimized synthetic procedures. Their comparably large dielectric constants not only raise the energetic cost of bis-chelate formation but also disfavor the association of the CIP required for the second chelate ligand to react and bind, keeping the individual ions separate until crystallization. Both factors direct the reaction toward selective isolation of the CIP rather than the bis-chelate. Conversely, conducting the synthesis in non-polar solvents favors CIP association already in solution while at the same time lowering the energetic cost required for the subsequent reaction to the bis-chelate, explaining why attempting the synthesis in chloroform, toluene, or dichloromethane yields intractable product mixtures.

### Cyclic voltammetry

The redox behavior of 1 and 2 was investigated by cyclic voltammetry (glassy carbon electrode, 100 mM [NBu_4_][PF_6_], 500 mV s^−1^; all potentials referenced to Fc^+^/Fc; Fig. 5).

**Fig. 5:**
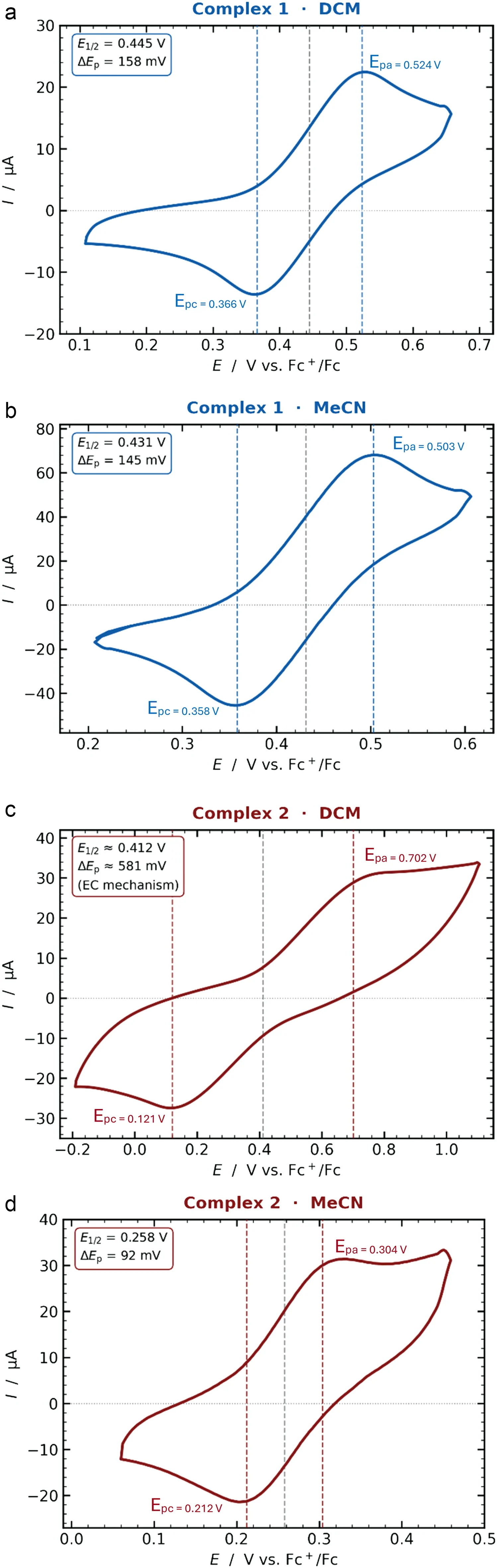
Cyclic voltammograms of complexes 1 (blue, panels a/b) and 2 (red, panels c/d) in DCM (a and c) and MeCN (b and d). Glassy carbon electrode, 500 mV s^−1^, 100 mM [NBu_4_][PF_6_]; all potentials *vs.* Fc^+^/Fc. Dashed vertical lines mark *E*_pa_, *E*^c^_p_ and *E*_1/2_.

Complex 1 shows a quasi-reversible Re(v)/Re(vi) oxidation in both solvents. In DCM, the redox event is centered at *E*_1/2_ = +0.445 V with a peak separation of Δ*E*_p_ = 158 mV. In MeCN, Δ*E*_p_ decreases to 145 mV and *E*_1/2_ shifts slightly to +0.431 V. The improved reversibility in MeCN is consistent with the higher donor number of acetonitrile (Gutmann DN = 14.1 *vs.* 1.0 for CH_2_Cl_2_), which stabilizes the transiently formed Re(vi) species.^31^

Complex 2 behaves markedly differently in each solvent. In CH_2_Cl_2_, the oxidation is quasi-irreversible with a broad anodic wave at *E*^a^_p_ = +0.702 V. The cathodic Re(vi)/Re(v) return wave is absent. Instead, a cathodic feature at *E*^c^_p_ = +0.121 V appears on the return scan, assigned to reduction of a secondary species formed by a rapid EC follow-up reaction, most likely chloride dissociation or solvent coordination at the oxidized Re(vi) center (Δ*E*_p_ ≈ 581 mV). In MeCN, this EC pathway is suppressed. Complex 2 shows a quasi-reversible couple centered at *E*_1/2_ = +0.258 V with a peak separation of Δ*E*_p_ = 92 mV.

The half-wave potentials in MeCN reveal a clear electronic substituent effect and show 2 (*E*_1/2_ = +0.258 V) is significantly easier to oxidize than 1 (*E*_1/2_ = +0.431 V) by 173 mV. This is consistent with the electron-donating methoxy group raising the electron density at the Re(v) center and is directly corroborated by the red-shifted IPCT band in the UV/Vis spectra of 2. The electrochemical ordering and spectroscopic trends thus converge on the same underlying electronic picture: the methoxy substituent facilitates the Re(v) → Re(vi) oxidation by increasing the electron density at the metal. A summary of all electrochemical data is given in Table 4.

**Table 4:** Cyclic voltammetric data for complexes 1 and 2 (*vs.* Fc^+^/Fc; GCE, 500 mV s^−1^)

Complex	Solvent	*E* ^a^_p_/V	*E* ^c^_p_/V	*E*_1/2_/V	Δ*E*_p_/mV
1	DCM	+0.524	+0.366	+0.445	158
1	MeCN	+0.503	+0.358	+0.431	145
2	DCM	+0.702	+0.121	≈+0.412	≈581 (EC)
2	MeCN	+0.304	+0.212	+0.258	92

### Catalytic perchlorate reduction

The catalytic activity of 1 and 2 towards perchlorate reduction, the benchmark reaction of this compound class, was evaluated using DMS (4 equiv.) as the terminal oxygen acceptor and [NBu_4_][ClO_4_] as substrate (10 mol% catalyst, CDCl_3_, room temperature, N_2_). Conversion was monitored by ^1^H NMR spectroscopy through the DMS/DMSO integral ratio (Table 5). The overall reaction is shown in Scheme 3. Conversions refer to the complete reduction of perchlorate to chloride, which consumes four equivalents of DMS per perchlorate. Turnover numbers and frequencies are therefore given per perchlorate reduced and not per oxygen atom transferred.

**Scheme 3:**
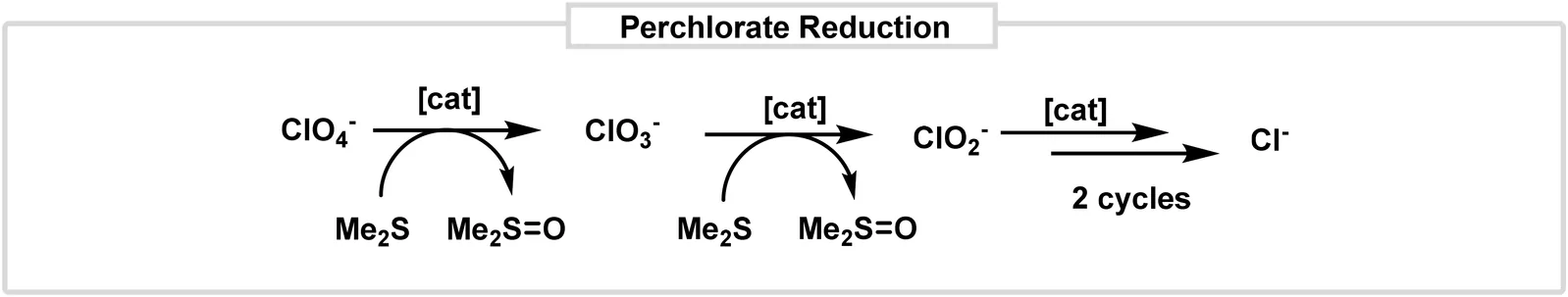
Overall equation of the catalytic perchlorate reduction.

**Table 5:** Catalytic perchlorate reduction data for complexes 1, 2 and 3

Complex	Load/mol%	Conditions	Conv./%	Time/h	TOF/h^−1^
1	10	Air, CDCl_3_	92	25	0.37
1	10	N_2_, CDCl_3_	92	25	0.37
2	10	N_2_, CDCl_3_	55	25	0.22
3	10	N_2_, CDCl_3_	90	25	0.36

Complex 1 achieves 92% conversion after 25 h (TOF = 0.37 h^−1^, Table 5), a result fully reproduced under nitrogen. The methoxy-substituted analogue 2 reaches only 55% conversion after 25 h (TOF = 0.22 h^−1^, Table 5). This reduced activity is unlikely to be a purely electronic ligand effect. The rate-determining step of perchlorate reduction is the oxygen atom transfer from perchlorate to Re(v), *i.e.* the oxidation of Re(v) to Re(vii).^1,3,4^ The more electron-rich Re(v) centre in 2, evidenced by the lower *E*_1/2_ in MeCN, should therefore facilitate rather than hinder this step. In line with this, electron-withdrawing substituents decrease the activity of bis-chelate complexes. The nitro-substituted [ReOCl(dmozNO_2_)_2_] reaches only 48% conversion compared with 75% for 3,^9^ and a dichloro-substituted analogue is even less active.^6^ Moreover, the methoxy group has practically no influence on the activity of the bis-chelate complexes (78% for 4*vs.* 75% for 3).^9^ The lower activity of 2 is therefore attributed mainly to differences in the formation of the catalytically active species from the ion pair, in line with the weaker cation–anion hydrogen bonding in 2. Consequently, electron-poor ion pair complexes are not expected to show an enhanced activity. Structure–activity relationships of related oxidorhenium(v) complexes have been discussed in detail elsewhere.^5,6,32^

To clarify the nature of the catalytically active species, the reaction of 1 was monitored by ^1^H NMR spectroscopy over the entire reaction time (Fig. S17, SI). The characteristic aromatic resonances of the bis-chelate complex 3 (*e.g. δ* 7.93 and 7.47 ppm) were not detected at any time, and the spectra of the catalytic mixture remained unchanged throughout the reaction. Conversion of 1 into 3 can therefore be excluded as the activation pathway. As a control, 3 was tested under identical conditions and reaches 90% conversion after 25 h (TOF = 0.36 h^−1^, Table 5). This activity is in line with that previously reported for 3.^9,32^ The spectra of the catalytic mixture differ from that of isolated 1, indicating that the ion pair is transformed into a distinct species under catalytic conditions, which persists during catalysis. The ion pair complex 1 therefore acts as a catalyst without prior conversion into the bis-chelate complex 3.

As established for related oxidorhenium(v) complexes, perchlorate reduction proceeds *via* two-electron oxygen atom transfer steps between Re(v) and Re(vii), without involvement of Re(vi).^1,3,4^ A detailed mechanistic investigation of perchlorate reduction by the ion pair complexes is beyond the scope of this work and is currently in progress.

## Conclusions

The oxidorhenate(v) outer-sphere ion pair complexes (H_2_L1)[ReOCl_3_(L1)] (1) and (H_2_L2)[ReOCl_3_(L2)] (2) have been synthesized, fully characterized in the solid state and in solution, and evaluated as perchlorate-reduction catalysts. Synthesis of 1 from [ReOCl_3_(SMe_2_)_2_] in acetonitrile gives 42% yield; competing formation of the fully coordinated analogue 3 is suppressed by short reaction times and a closed reaction vessel that prevents HCl loss. X-ray diffraction confirms that both compounds crystallize as contact ion pairs stabilized by networks of N–H⋯Cl and O–H⋯Cl hydrogen bonds. In 1, the hydrogen-bond-accepting Re–Cl bonds are elongated by up to 0.055 Å relative to the non-hydrogen-bonded *trans* chlorido ligand, indicating pre-activated lability consistent with the catalytic competence of the complex; the weaker cation–anion hydrogen bonding in 2 results in a markedly reduced bond-length spread of only 0.015 Å.

UV/Vis spectroscopy across a polarity series shows that the intense purple coloration of 1 and 2 in apolar media originates from an ion pair charge transfer (IPCT) transition, assigned to formal electron transfer from the Re(v) d_*xy*_ HOMO of [ReOCl_3_(ON)]^−^ to the π* orbital of the H_2_(ON)^+^ iminium fragment, mediated by the hydrogen-bond network. The methoxy substituent in 2 red-shifts the IPCT band by 22 nm and reduces *ε* about threefold, consistent with a raised Re(v) d-manifold energy and weakened inter-ionic electronic coupling.

DFT calculations across a series of solvents reveal that formation of the contact ion pair and subsequent bis-chelate association is strongly favored in apolar media, while increasing solvent polarity progressively destabilizes both species and renders bis-chelate formation particularly unfavorable relative to the CIP. This thermodynamic picture reinforces the UV/Vis evidence for CIP association in apolar solvents and dissociation in polar ones, and provides a rationale for why the optimized syntheses in acetonitrile and acetone selectively afford the CIP, whereas reactions in apolar solvents yield product mixtures.

Cyclic voltammetry in MeCN shows that 2 (*E*_1/2_ = +0.258 V) is 173 mV easier to oxidize than 1 (*E*_1/2_ = +0.431 V *vs.* Fc^+^/Fc), confirming that the methoxy group raises electron density at the Re(v) center; the EC-type irreversible response of 2 in DCM independently corroborates the solvent-dependent ion pair geometry. In perchlorate reduction, 1 and 2 act as catalysts, and ^1^H NMR monitoring shows that 1 is not converted into the bis-chelate complex 3 during catalysis. The lower activity of 2 (55% *vs.* 92% conversion after 25 h) cannot be explained by the electronic effect of the methoxy group alone and is attributed mainly to differences in the formation of the catalytically active species.

## Experimental section

### General remarks

All reactions were performed under nitrogen using standard Schlenk techniques unless otherwise stated. Solvents were dried on a Pure Solv® system and stored over molecular sieves. ^1^H and ^13^C{^1^H} NMR spectra were recorded on a Bruker Avance® III (300 MHz) spectrometer and referenced to residual solvent signals (CDCl_3_: *δ*^1^H 7.26, *δ*^13^C 77.16; CD_3_CN: *δ*^1^H 1.94, *δ*^13^C 118.26). Multiplicities: s (singlet), bs (broad singlet), d (doublet), t (triplet), dd, ddd, m. ATR-IR spectra were measured on a Bruker ALPHA® FT-IR spectrometer (diamond probe); intensities: s (strong), m (medium), w (weak). UV/Vis spectra were recorded on a Varian Cary® 50 spectrophotometer (1 cm quartz cuvette, rt). EI-MS spectra were obtained on an Agilent 5973 MSD direct probe (70 eV). Single-crystal X-ray data for 1 were collected with Mo Kα radiation (*λ* = 0.71073 Å) at 100 K; data for 2 on a XtaLAB Synergy, Dualflex, HyPix-Arc 100 diffractometer, Mo Kα, 100 K. Structures solved (SHELXT) and refined on *F*^2^ (SHELXL-2019/3). Cyclic voltammetry: Gamry REF600 potentiostat, glassy carbon working electrode (3 mm), Pt-wire counter, Ag-wire pseudo-reference, 100 mM [NBu_4_][PF_6_] supporting electrolyte, 500 mV s^−1^; potentials *vs.* Fc^+^/Fc (internal ferrocene standard). DFT: TURBOMOLE v7.9, ωB97X-D3/dhf-TZVPP//PBE-D3BJ/dhf-SVP, COSMO (see Computational Details). Hdmoz (HL1),^33^ HdmozOMe (HL2),^34^ [ReOCl_3_(SMe_2_)_2_] (P2)^35^ and [ReOCl_3_(OPPh_3_)(SMe_2_)] (P1)^36,37^ were prepared following published procedures. The authors declare that generative AI tools were used to assist with language editing and data analysis in the preparation of this manuscript.

### Synthesis of (H_2_L1)[ReOCl_3_(L1)] (1)

[ReOCl_3_(SMe_2_)_2_] (P2; 0.43 g, 1.00 mmol, 1.0 equiv.) and Hdmoz (HL1; 0.38 g, 2.00 mmol, 2.0 equiv.) were suspended in dry MeCN (30 mL) in a sealed 100 mL Schlenk flask under N_2_. The mixture was heated to 60 °C for 1 h, cooled to room temperature, and stirred for 2 h. The solvent was evaporated *in vacuo*. The residue was washed with EtOAc (6 × 2 mL) until the washings turned brownish; the light green solid was dried *in vacuo*. Yield: 0.29 g (0.42 mmol, 42%). Recrystallisation from concentrated MeOH afforded crystals suitable for X-ray diffraction. The use of acetone instead of MeCN as reaction solvent gave the same result. ^1^H NMR (300 MHz, CDCl_3_): *δ* 7.79 (dd, *J* = 8.1, 1.6 Hz, 2H, Ar), 7.61 (ddd, *J* = 8.1, 7.1, 1.6 Hz, 2H, Ar), 7.39 (d, *J* = 8.4 Hz, 2H, Ar), 7.02 (t, *J* = 7.6 Hz, 2H, Ar), 4.68 (s, 4H, –CH_2_^−^), 1.72 (s, 12H, CH_3_). ^13^C NMR (75 MHz, CDCl_3_): *δ* 139.66, 130.38, 120.87, 118.95, 117.74, 82.27, 63.58, 27.77. ^13^C NMR (75 MHz, CD_3_CN): *δ* 169.42, 165.89, 160.66, 139.82, 137.55, 132.05, 131.85, 121.81, 117.97, 109.19, 107.47, 83.02, 79.53, 74.46, 64.46, 26.62, 26.54. Owing to the low solubility of 1, not all ^13^C signals could be detected in CDCl_3_. ATR-IR (*ν̃*/cm^−1^): 3120 (w), 2985 (w), 1609 (s, C=N), 1481 (m), 1377 (m), 1267 (m), 1085 (s), 974 (s, Re=O), 881 (m), 743 (s), 692 (m). UV/Vis [*λ*_max_/nm (*ε*/L mol^−1^ cm^−1^)]: MeCN: 668 (49); CHCl_3_: 555 (2700); EtOAc: 531 (1100). EI-MS (*m*/*z*): 709 (M + NH_4_^+^), 640 (M^+^–O–Cl), 484 (M^+^–H_2_L1–O).

### Synthesis of (H_2_L2)[ReOCl_3_(L2)] (2)

[ReOCl_3_(OPPh_3_)(SMe_2_)] (P1; 0.65 g, 1.00 mmol, 1.0 equiv.) and HdmozOMe (HL2; 0.47 g, 2.20 mmol, 2.2 equiv.) were dissolved in acetone (200 mL) and refluxed for 5 h. The solution was concentrated *in vacuo* to ∼10 mL and added dropwise to Et_2_O (100 mL); the green precipitate was filtered and dried *in vacuo*. Recrystallisation from concentrated acetone gave 2 as an emerald-green solid (39%, 0.29 g, 0.39 mmol). Note: recrystallisation from EtOH/iPrOH yields [ReOCl(L2)_2_] (4) instead. The use of MeCN instead of acetone as reaction solvent gave the same result. ^1^H NMR (300 MHz, CDCl_3_): *δ* 7.36–7.32 (m, 2H, Ar), 7.06 (dd, 1H, Ar), 6.93–6.79 (m, 3H, Ar), 4.64 (s, 2H, CH_2_), 4.55 (s, 2H, CH_2_), 3.73 (s, 3H, OCH_3_), 3.62 (s, 3H, OCH_3_), 1.81 (s, 6H, CH_3_), 1.74 (s, 6H, CH_3_). ^13^C NMR (75 MHz, CDCl_3_): *δ* 148.23, 124.12, 120.81, 120.26, 82.57, 65.50, 64.17, 27.56. Owing to the low solubility of 2, not all ^13^C signals could be detected. ATR-IR (*ν̃*/cm^−1^): 3181 (w), 2971 (w), 1607 (s, C=N), 1505 (m), 1256 (s), 1055 (s), 970 (s, Re=O), 869 (m), 693 (s). UV/Vis [*λ*_max_/nm (*ε*/L mol^−1^ cm^−1^)]: MeCN: 705 (57); CHCl_3_: 577 (920); EtOAc: 572 (680). EI-MS (*m*/*z*): 700 (M^+^–O–Cl), 493 (M^+^–H_2_L2–Cl).

### X-ray crystal structure determination

Crystal data for 1: C_11_H_12_Cl_3_NO_3_Re·C_11_H_14_NO_2_, *M*_r_ = 691.00, monoclinic, *P*2_1_/*c*, *a* = 10.6059(5) Å, *b* = 12.2574(6) Å, *c* = 19.0613(9) Å, *β* = 99.844(2)°, *V* = 2441.5(2) Å^3^, *Z* = 4, Mo Kα, 100 K; *R*_1_ = 0.0256, w*R*_2_ = 0.0557, GOF = 1.051. Crystal data for 2: C_24_H_30_Cl_3_N_2_O_7_Re, *M*_r_ = 751.05, orthorhombic, *P*2_1_2_1_2_1_ (No. 19), *a* = 9.1936(2) Å, *b* = 16.7648(3) Å, *c* = 17.9504(3) Å, *V* = 2766.67(9) Å^3^, *Z* = 4, Flack = −0.019(4); *R*_1_ = 0.0286, w*R*_2_ = 0.0584, GOF = 1.009. CCDC 2574891 (1) and 2574892 (2) contain the supplementary crystallographic data. Full crystal data are given in Table S5 (SI).

### Computational details

Geometry optimizations for the contact ion-pair complexes 1–4 were performed at the PBE-D3BJ/dhf-SVP level of theory using the COSMO solvation model, with single-point energies and thermochemical corrections evaluated at the ωB97X-D3/dhf-TZVPP level in all eight solvents at 25 °C and 1 bar.

### Catalytic perchlorate reduction

The catalyst (10 mol%), [NBu_4_][ClO_4_] (1.0 equiv.) and DMS (4.0 equiv.) were dissolved in CDCl_3_ (0.5 mL) in a J-Young NMR tube under N_2_. Conversion was monitored by ^1^H NMR spectroscopy through the DMSO (*δ* 2.55): DMS (*δ* 2.13) integral ratio. TOF values were calculated at the point of maximum DMSO yield.

## Author contributions

T. A. D.: investigation, data curation, formal analysis, writing – original draft. A. M. S.: investigation, data curation. P. E. H., A. D. B., K. K., M. M., F. B., A. D.: formal analysis, writing – original draft. J. A. S.: conceptualization, funding acquisition, supervision, writing – review & editing.

## Conflicts of interest

There are no conflicts to declare.

## Supplementary Material

DT-055-D6DT02275H-s001

## Data Availability

Details regarding X-ray data collection and DFT calculations as well as NMR data of novel compounds are available within the article and its supplementary information (SI). Metadata is deposited at Zenodo under https://doi.org/10.5281/zenodo.22812492. Supplementary information is available. The SI contains the reaction condition screening (Table S1), solvatochromism and UV/Vis data (Tables S2 and S3, Fig. S1–S7), catalytic data (Table S4, Fig. S8 and S17), NMR spectra (Fig. S9–S16), crystallographic data and molecular structures (Tables S5–S7, Fig. S18 and S19), and computational details and DFT energies (Tables S8–S12). See DOI: https://doi.org/10.1039/d6dt02275h. CCDC 2574891 and 2574892 (1 and 2) contain the supplementary crystallographic data for this paper.^38*a*,*b*^
