# Supplementary material for: Ion-pair complexes of oxidorhenate(v) with phenolate–oxazoline ligands: synthesis, solvatochromism and catalytic properties
Source: Dalton Trans. 2026 Oct 27;55(43):15564–74. doi: 10.1039/d6dt02275h (PMC13712253; doi:10.1039/d6dt02275h)
Supplement: Supplementary file 2 [file DT-055-D6DT02275H-s001.pdf]

Supplementary Information (SI) for Dalton Transactions.
This journal is © The Royal Society of Chemistry

Electronic Supplementary Information (ESI)

Ion-Pair Complexes of Oxidorhenate(V) with Phenolate–Oxazoline Ligands: Synthesis, Solvatochromism and Catalytic Properties

Tobias A. Doliner,[a] Anna M. Schlegl,[a] Kristin Kogler,[a] Milan Milovanović,[a,b] A. D. Boese,[a] Ferdinand Belaj,[a] Antoine Dupé,[a] Peter E. Hartmann[a] and Jörg A. Schachner[a,□]

□ *Institute of Chemistry, University of Graz, Schubertstraße 1, 8010 Graz, Austria.*

[b] *Innovation Center of the Faculty of Chemistry, Belgrade, Studentski trg 12-16, 11158 Belgrade, Serbia.*

corresponding author: joerg.schachner@uni-graz.at

Table S1 Reaction condition screening for the synthesis of $(H_2dmoz)[ReOCl_3(dmoz)]$ (**1**)

Solvent	T [°C]	After 2 h	After 15 h
Acetonitrile	60	Mainly **1**; minor unidentified side product	Less **1** than after 2 h; more side product
Acetonitrile	rt	Mixture **1**, **3** and isomers	No significant change
Acetonitrile	60 then rt	Mainly **1**, traces of side product	No significant change
Chloroform	60	Mixture **1**, **3** or isomers; side products	No significant change
Chloroform	rt	Some **1** and **3**; mainly unidentified side product	No significant change
Toluene	60	Some **1**; multiple unidentified side products	Some **1**; possible isomer of **1**
Toluene	rt	No significant **1**	No significant change
Dichloromethane	35	Some **1** and side products	More **3**; some **1**; side products
Dichloromethane	rt	No target complex	No significant change

Table S2 Observed color of complexes **1** and **2** in various solvents

Solvent	Complex 1	Complex 2	Z [kcal mol−1]a	εr b
Cyclohexane	insoluble	insoluble	—	2.0
Toluene	insoluble	insoluble	—	2.4
Diethyl ether	insoluble	insoluble	—	4.3
Chloroform	purple	purple	63.2	4.8
Ethyl acetate	purple	purple	64.0	6.0

Dichloromethane	greenish purple	purple	64.2	9.1
1,2-Dichloroethane	greenish purple	purple	63.4	10.6
Acetone	green	purple	65.7	20.7
Ethanol	green	green	79.6	24.3
Methanol	green	green	83.6	33.6
Acetonitrile	green	green	71.3	37.5

Table S3 UV/Vis absorption parameters for complexes **1** and **2** in all investigated solvents

Solvent	λmax (1) / nm	ε (1) / L mol−1 cm−1	λmax (2) / nm	ε (2) / L mol−1 cm−1
Acetonitrile	668	49	705	57
Chloroform	555	2700	577	920
Ethyl acetate	531	1100	572	680

ε values in chloroform and ethyl acetate taken at or near maximum absorbance to account for kinetically controlled ion pair formation. Concentration range: 0.5–1.0 mmol L^{-1}. Time to reach maximum absorbance: ~11 h in $CHCl_3$; ~12 min in EtOAc for complex 2.

Table S4 Catalytic perchlorate reduction: full dataset for complexes **1**, **2** and **3**

Complex	Load. [mol%]	Conditions	Conv. [%]	Time [h]	TOF [h−1]
1	10	Air, CDCl3	92	25	0.37
1	10	N2, CDCl3	92	25	0.37
2	10	N2, CDCl3	55	25	0.22
**3**	10	N_2, $CDCl_3$	90	25	0.36

Substrate: NBu_4ClO_4 ($CDCl_3$) or $LiClO_4$ (CD_3CN/D_2O); DMS (4 equiv.); rt. TOF at maximum conversion. Value in parentheses after 48 h.

UV/Vis spectra

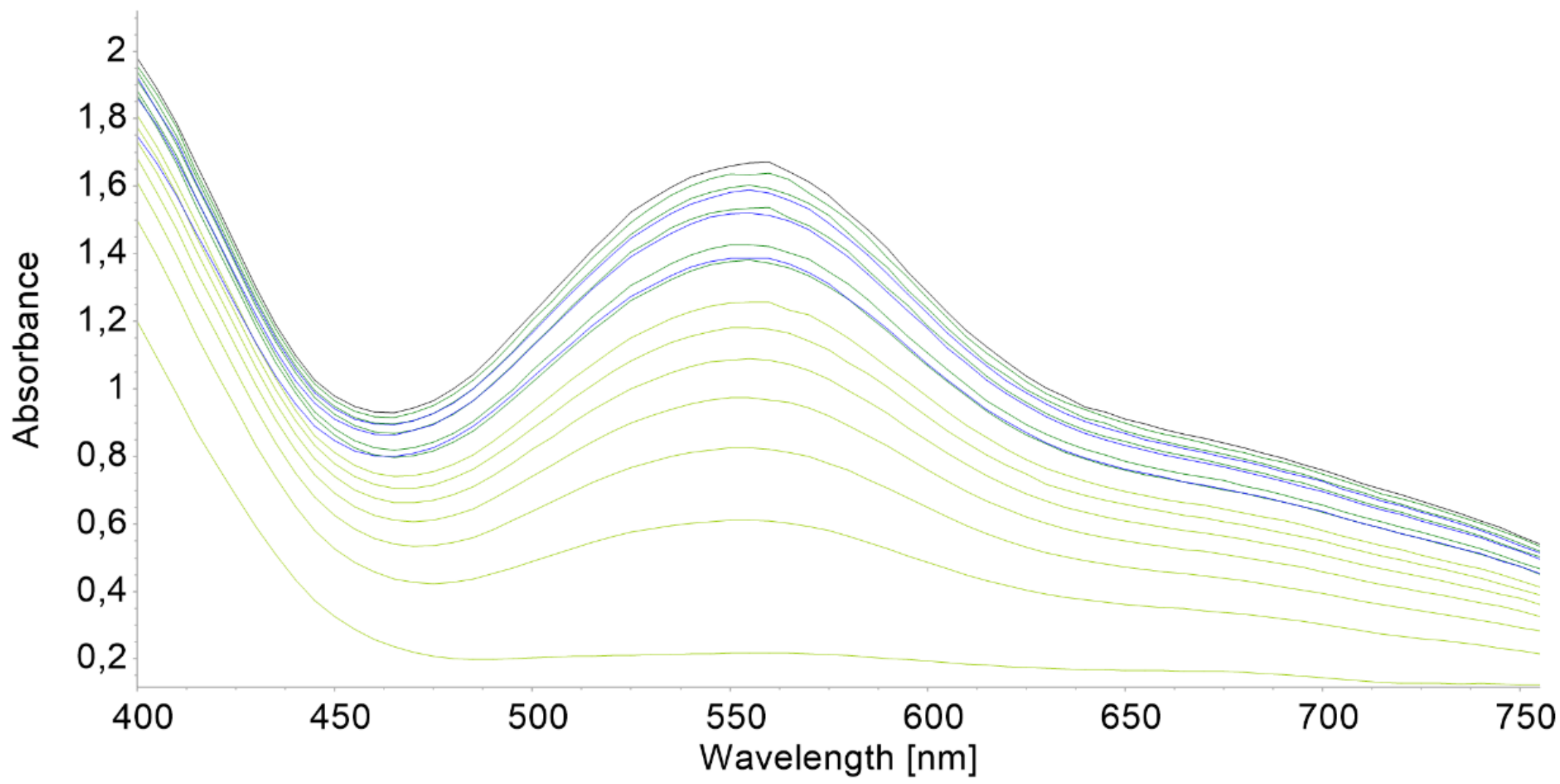

Fig. S1 UV/Vis spectral time series of complex **1** in chloroform (0–24 h). UV/Vis spectra of $(H_2dmoz)[ReOCl_3(dmoz)]$ (**1**; 0.61 mmol L^{-1}) in $CDCl_3$ recorded over 24 h. Light green traces (h 1–4): initial spectra showing gradual growth of the IPCT band at $λ_{max}$ = 555 nm. Dark green traces (h 5–10): continued absorbance increase. Grey trace (~12.5 h): near-maximum absorbance (ε = 2700 L mol^{-1} cm^{-1}). Blue traces (h 16–24): progressive decrease in absorbance accompanied by NMR evidence for conversion of **1** to **3** and decomposition products. The growth phase reflects kinetically controlled contact ion pair formation; the decay reflects complex decomposition.

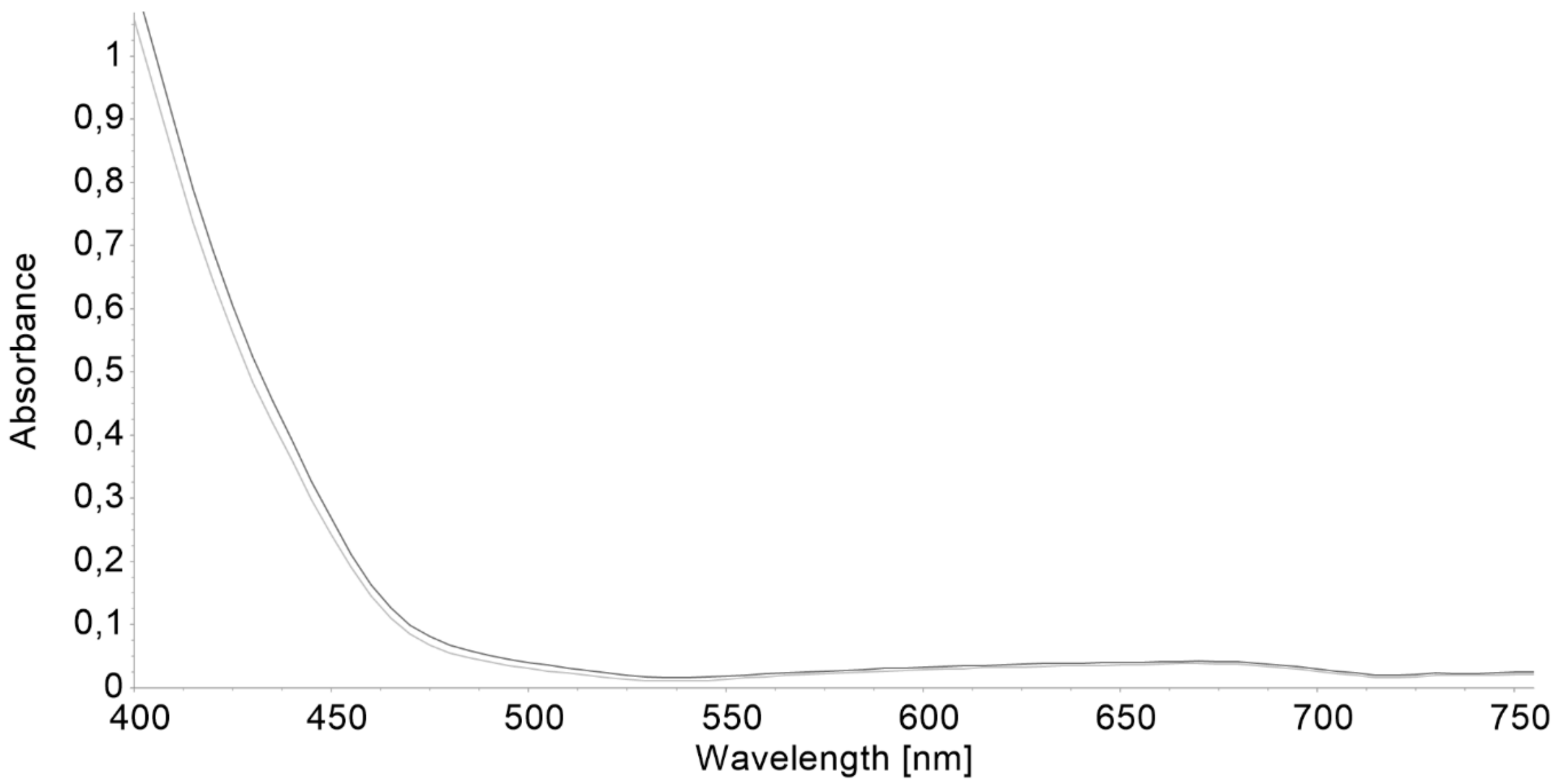

Fig. S2 UV/Vis spectra of complex **1** in acetonitrile. UV/Vis spectra of **1** (0.80 mmol L^{-1}) in CD_3CN. Black trace: 1 min after dissolution; grey trace: 60 min after dissolution. The two spectra are superimposable, confirming that no time-dependent spectral change occurs in this polar solvent. $λ_{max}$ = 668 nm; ε = 49 L mol^{-1} cm^{-1}. The weak, time-independent absorption is assigned to the Laporte-forbidden d–d transition of the effectively dissociated $[ReOCl_3(dmoz)]^-$ anion.

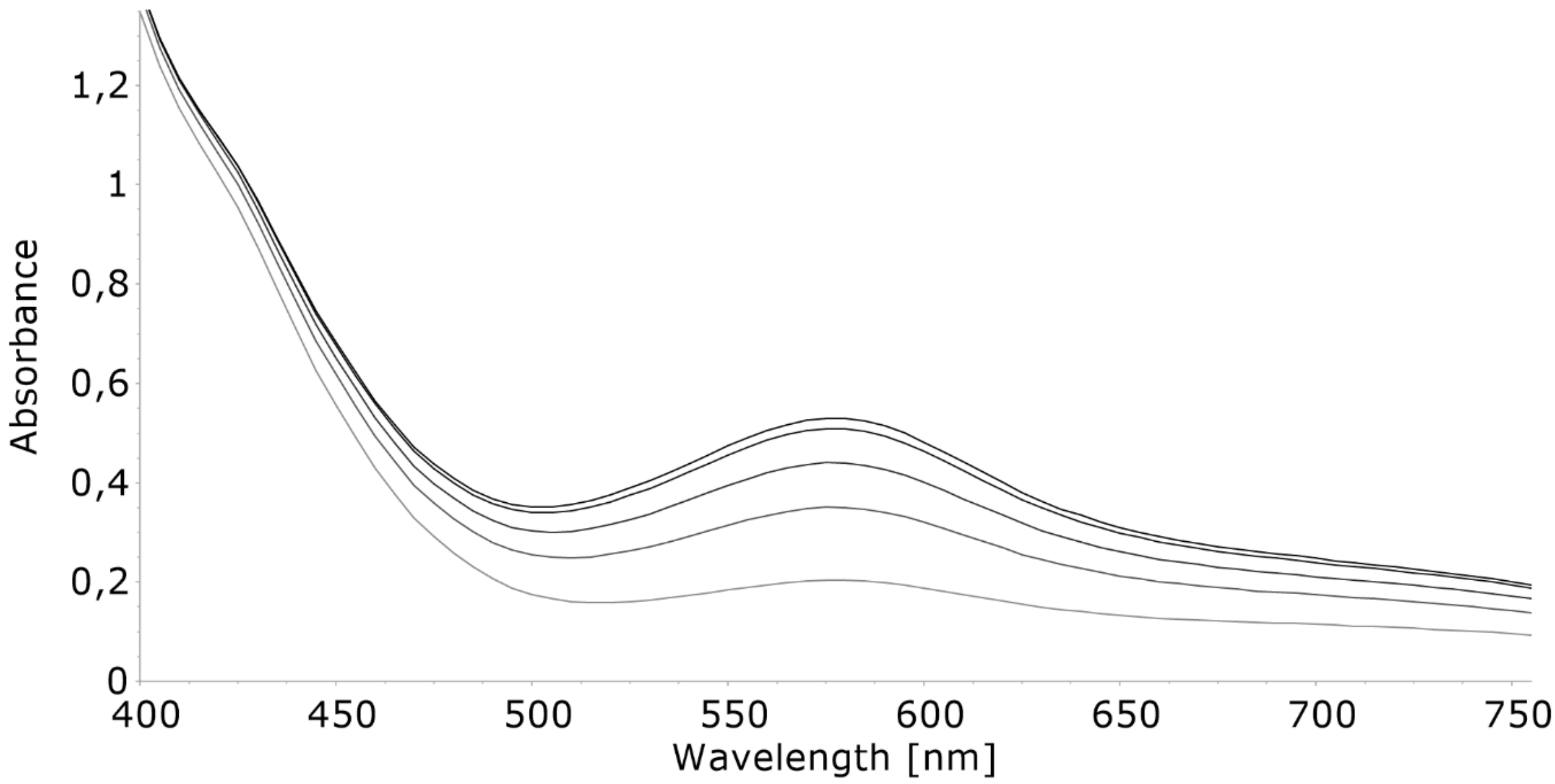

Fig. S3 UV/Vis time series of complex **2** in chloroform. UV/Vis spectra of (H_2dmozOMe)[ReOCl_3(dmozOMe)] (**2**; 1.0 mmol L^{-1}) in $CDCl_3$ after 1, 15, 30, 50, and 60 min. The IPCT band appears at $λ_{max}$ = 577 nm (ε = 530 L mol^{-1} cm^{-1} after 60 min; maximum ε = 920 L mol^{-1} cm^{-1} after about 2 h, **Fig. S4**). No shift in $λ_{max}$ is observed over the measurement period, in contrast to the behavior in ethyl acetate (**Fig. S6**). The lower ε compared to **1** in $CDCl_3$ (920 vs. 2700 L mol^{-1} cm^{-1}) is discussed in the main text in terms of reduced donor–acceptor electronic coupling in **2**.

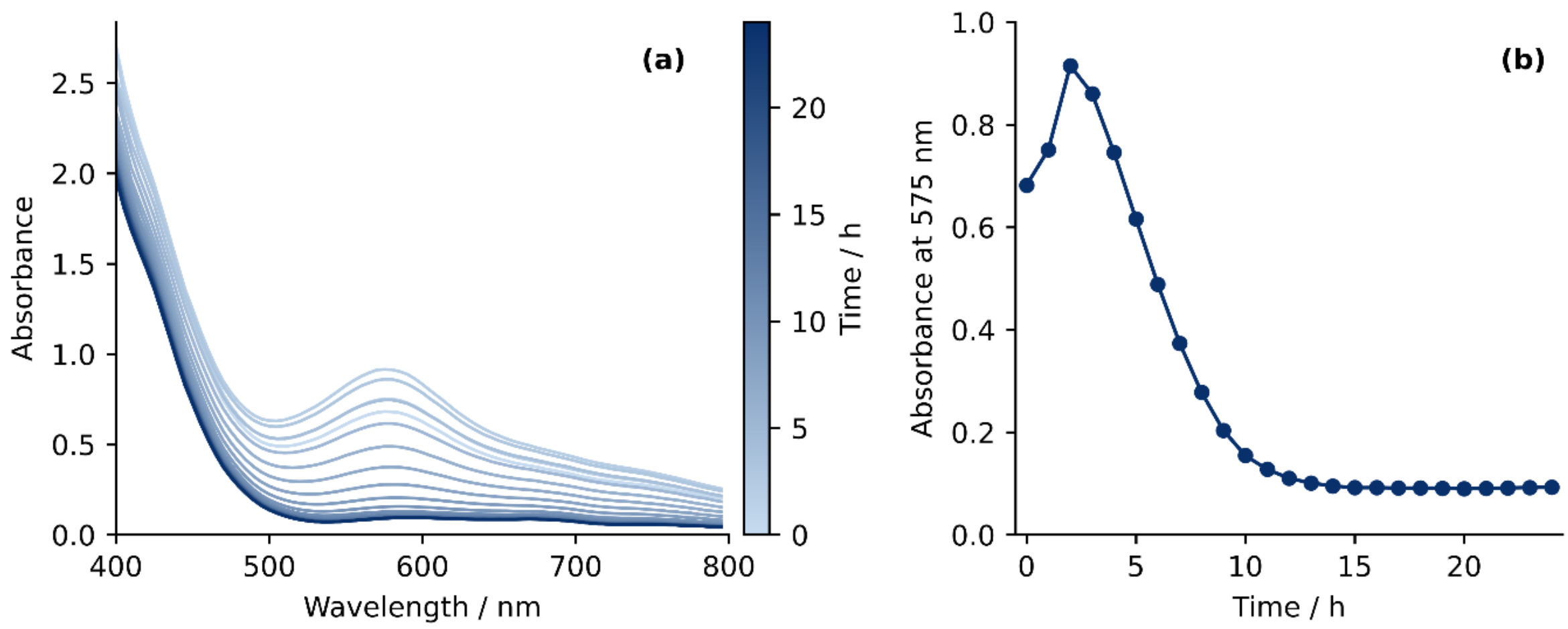

Fig. S4 UV/Vis long-term measurement of complex **2** in chloroform. UV/Vis spectra of (H_2L2)[ReOCl_3(L2)] (**2**; c = 1.0 mmol L^{-1}) in $CHCl_3$ recorded every hour over 24 h (a) and time dependence of the absorbance at 575 nm (b). The IPCT band at 575 nm reaches its maximum after about 2 h and decreases to about 10% of this value within 12 h. Maximum ε = 920 L mol^{-1} cm^{-1}.

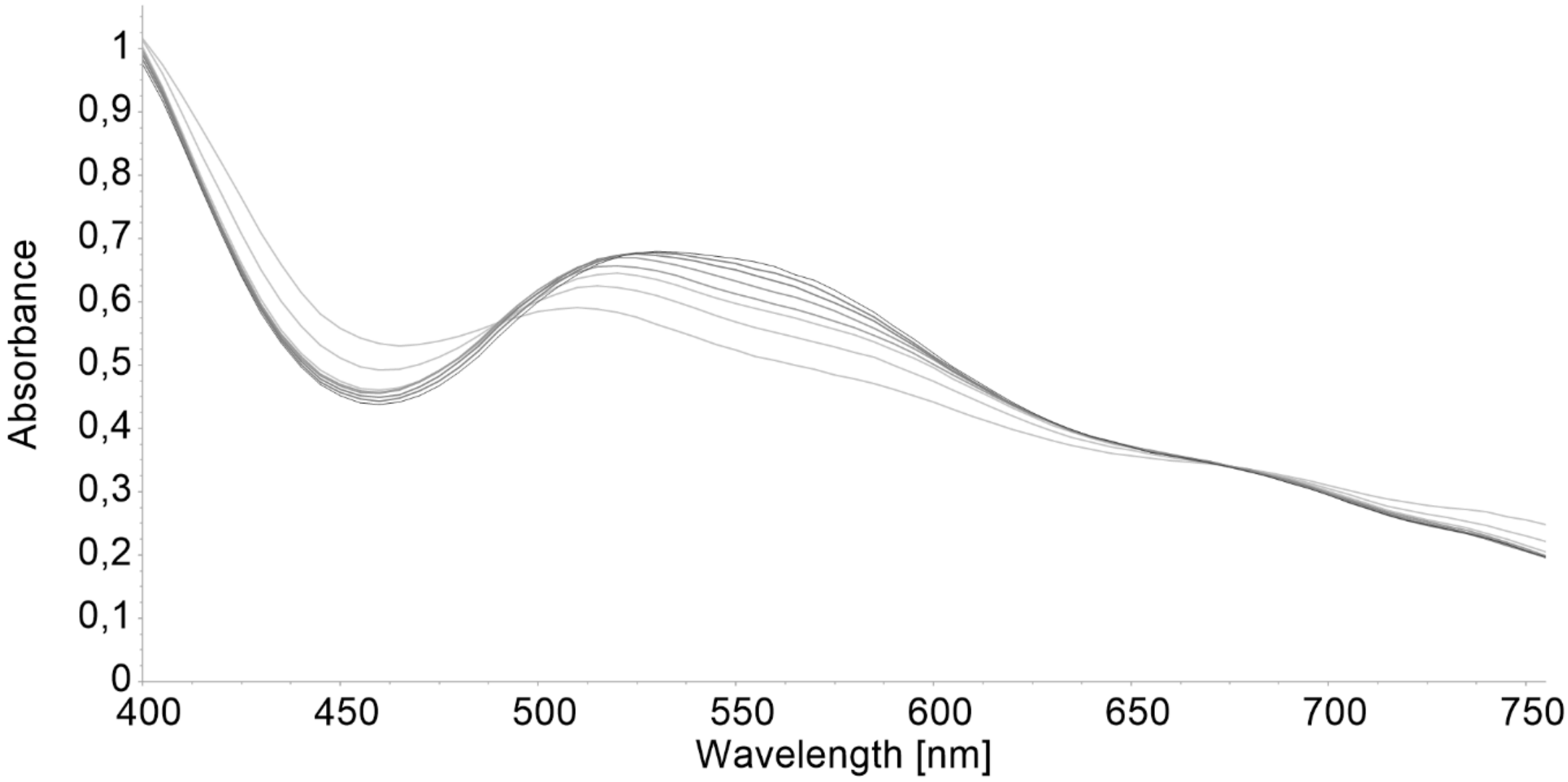

Fig. S5 UV/Vis time series of complex **1** in ethyl acetate. UV/Vis spectra of **1** (0.61 mmol L^{-1}) in ethyl acetate recorded over 105 min. Both the absorbance and λ$_{max}$ evolve with time: λ$_{max}$ shifts from 510 nm (initial) to 531 nm (105 min) with a simultaneous increase in ε to 1100 L mol^{-1} cm^{-1}. This concurrent bathochromic shift and intensity increase is attributed to the progressive formation of tighter contact ion pairs with increasing donor–acceptor proximity and is absent in chloroform where CIP formation occurs more rapidly.

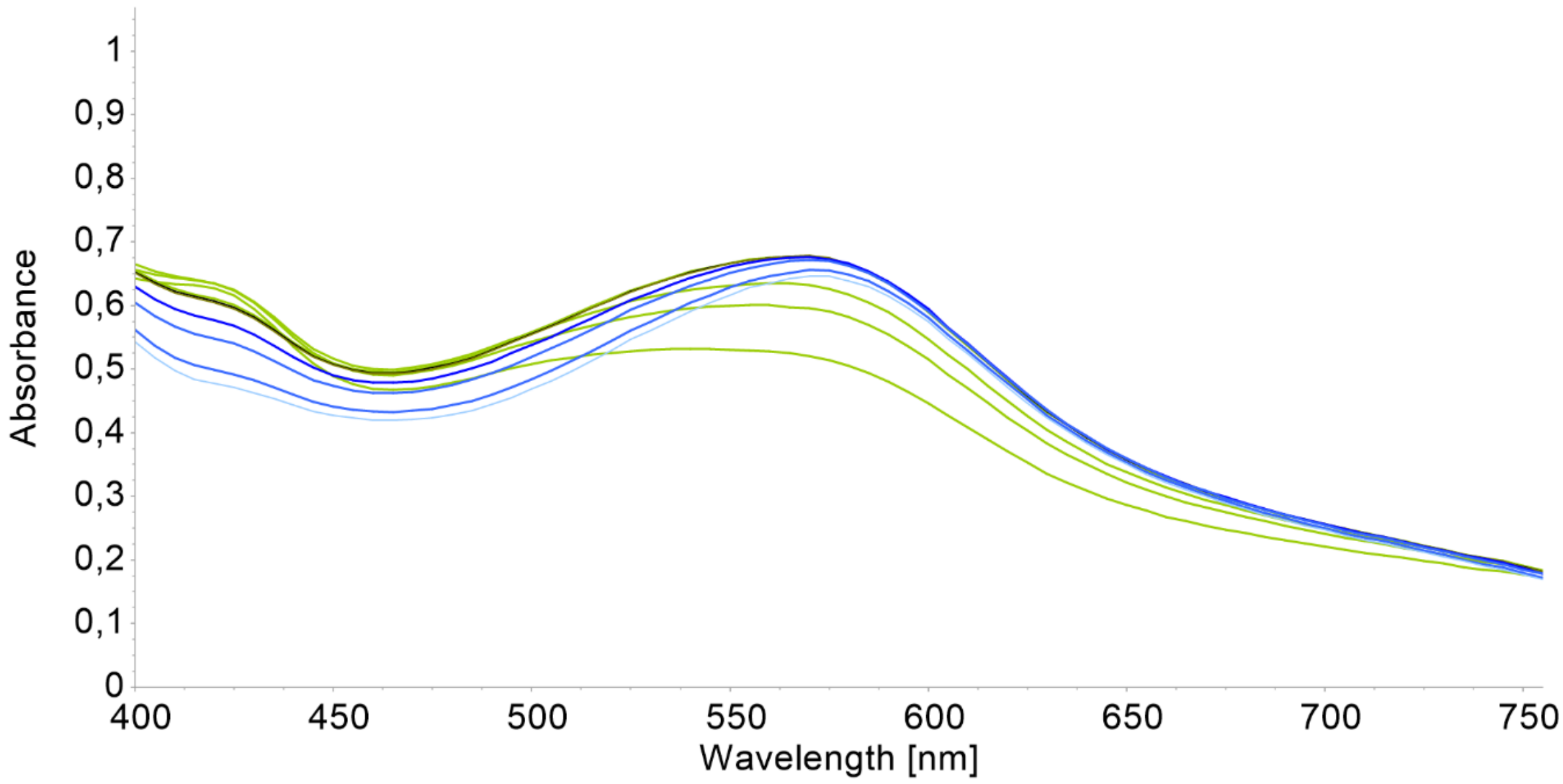

Fig. S6 UV/Vis time series of complex **2** in ethyl acetate. UV/Vis spectra of **2** (1.0 mmol L^{-1}) in ethyl acetate. Green traces: 1, 2, 3, and 10 min after dissolution; black: 20 min; blue: 30, 40, 50 min; light blue: 60 min. The IPCT band shifts from 539 nm (initial) to 572 nm over 60 min, with most of the spectral change occurring within the first 12 min. The final ε = 680 L mol^{-1} cm^{-1} is lower than the maximum value in chloroform (920 L mol^{-1} cm^{-1}), consistent with less complete CIP formation in this moderately polar solvent.

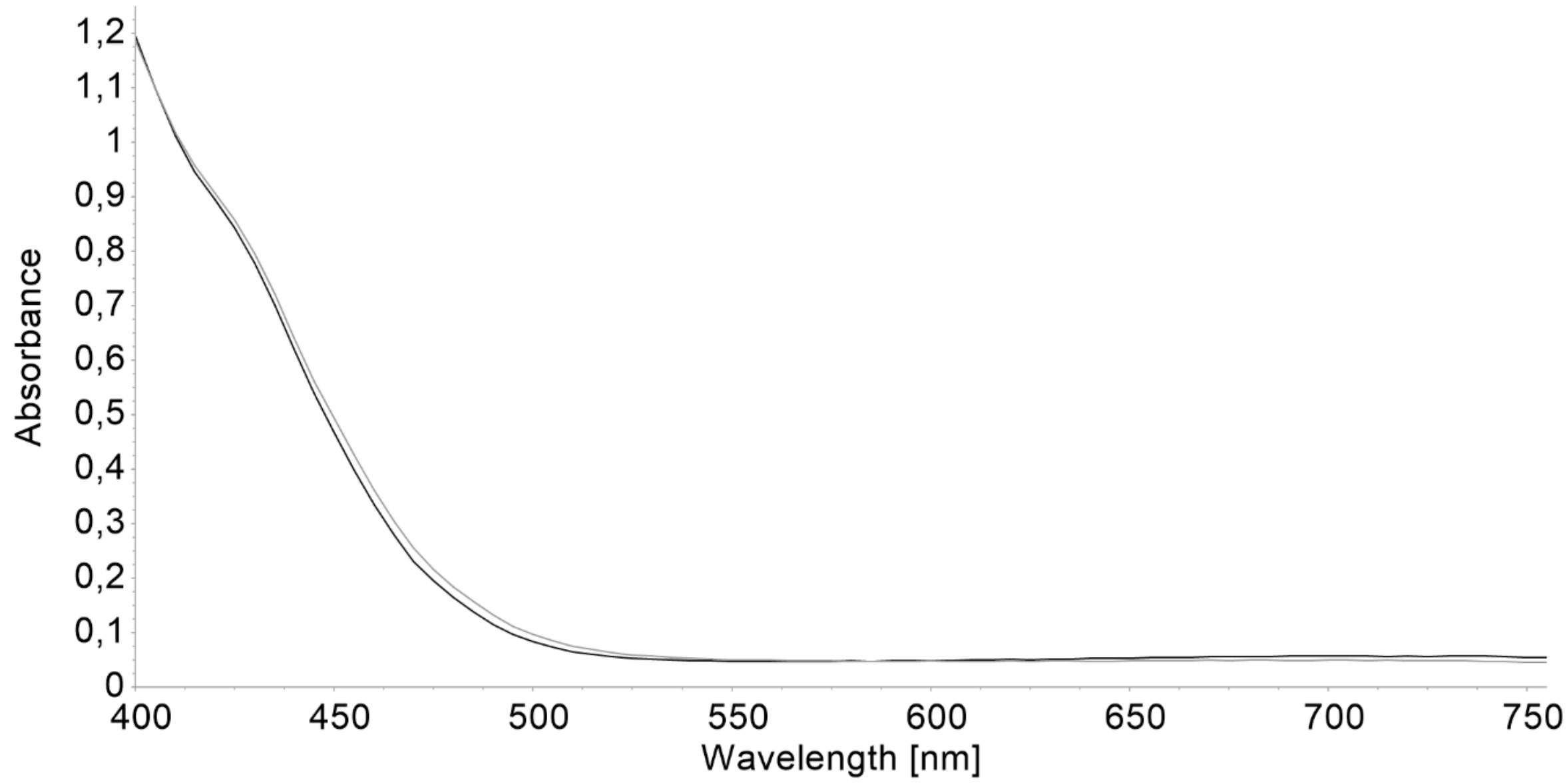

Fig. S7 UV/Vis spectra of complex **2** in acetonitrile. UV/Vis spectra of **2** (0.99 mmol L^{-1}) in CD_3CN. Black trace: 1 min; grey trace: 60 min after dissolution. λ$_{max}$ = 705 nm; ε = 57 L mol^{-1} cm^{-1}. As for **1**, no time dependence is observed, confirming complete absence of IPCT in the polar solvent. The red-shift of 37 nm relative to **1** (705 vs. 668 nm) is attributed to the elevated Re(V) d-manifold energy imparted by the electron-donating methoxy group (see main text).

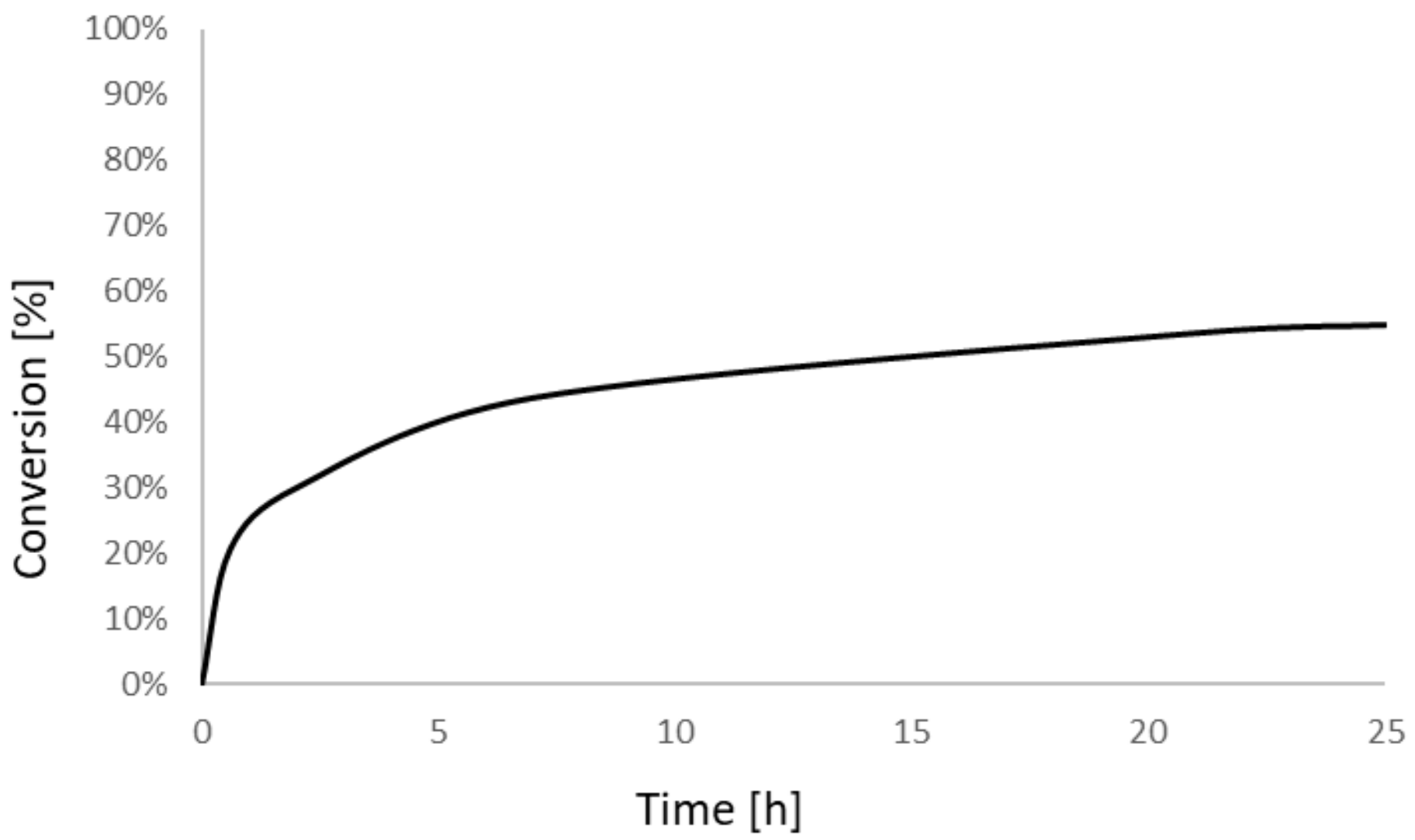

Fig. S8 Time-conversion plots: perchlorate reduction by complex **2**. Time-conversion plot for reduction of NBu_4ClO_4 (1.0 equiv.) by **2** (10 mol%) in $CDCl_3$ under N_2, monitored by conversion of DMS to DMSO (^{1}H NMR). Maximum conversion: 55% after 25 h. For comparison, **1** achieves 92% under the same conditions.

NMR Spectral Data

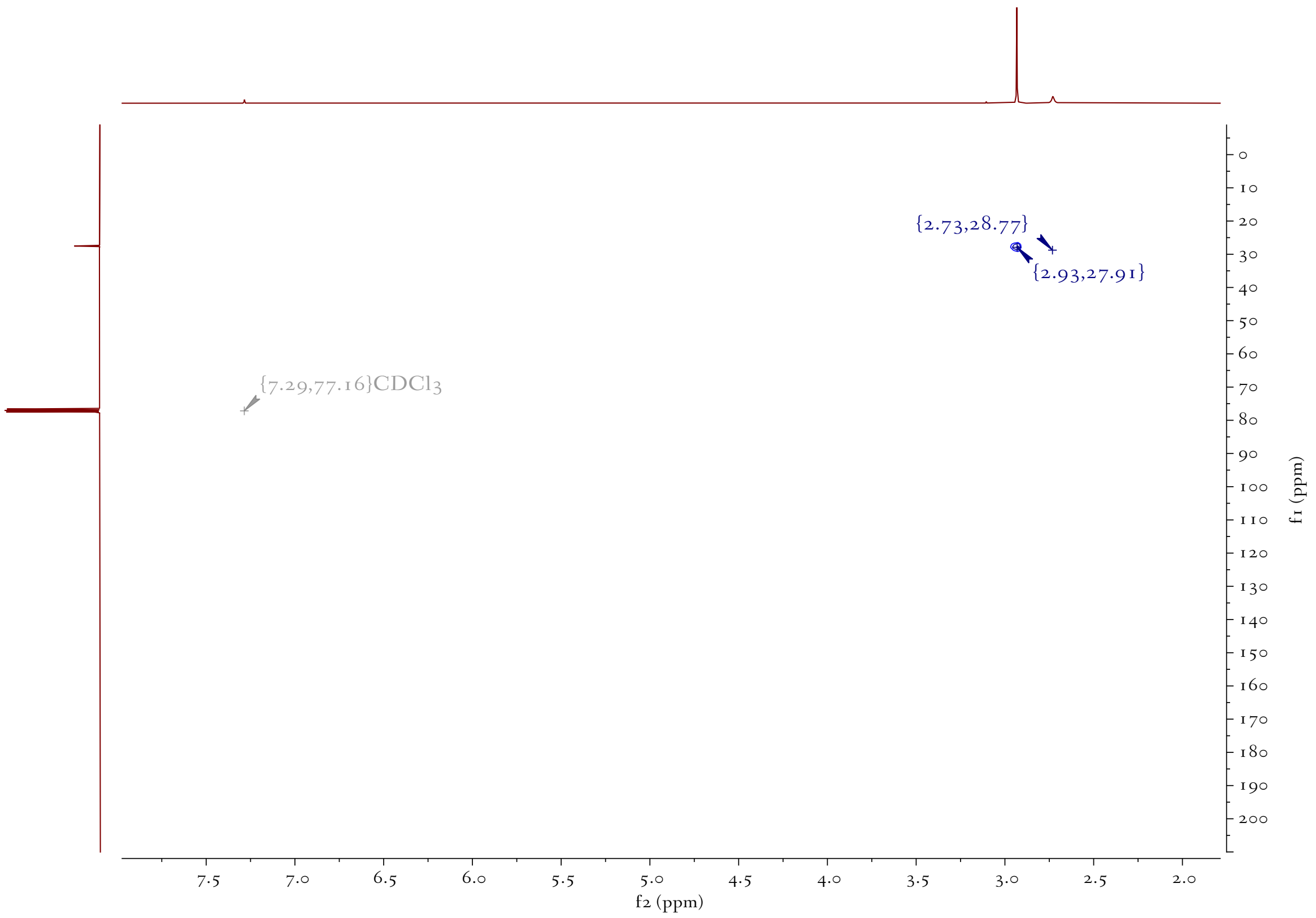

Fig. S9 HSQC of complex **1** in $CDCl_3$

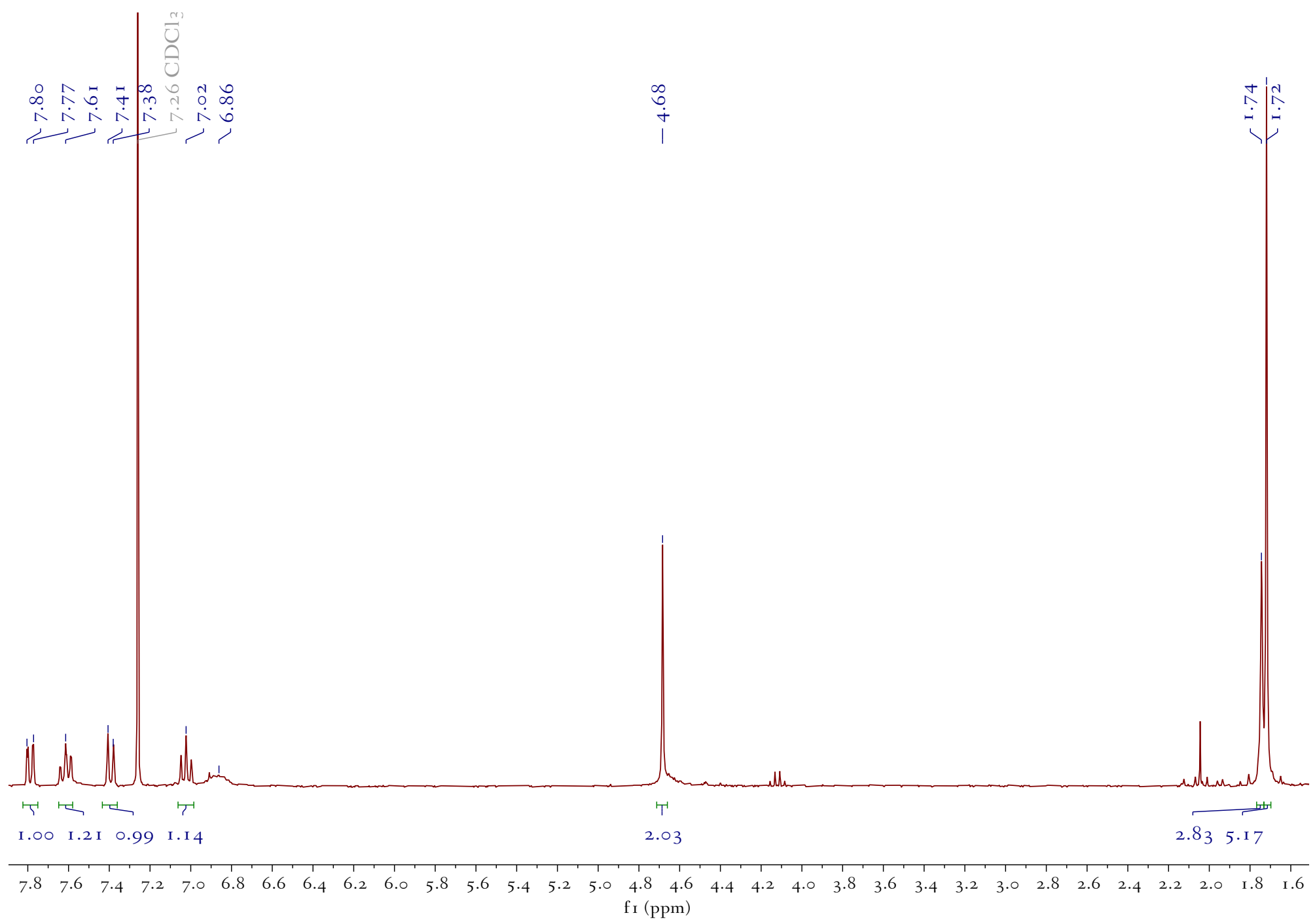

Fig. S10 Ion Pair Complex **1** in $CDCl_3$. ^{1}H NMR (300 MHz, $CDCl_3$): δ 7.79 (dd, *J* = 8.1, 1.6 Hz, 2H, H-6 Ar), 7.61 (ddd, *J* = 8.1, 7.1, 1.6 Hz, ~2H, H-4 Ar), 7.39 (d, *J* = 8.4 Hz, 2H, H-3 Ar), 7.02 ("t", *J* = 7.6 Hz, 2H, H-5 Ar), 4.68 (s, ~4H, $–CH_2–$), 1.72 (s, ~12H, CH_3). In $CDCl_3$ the proton exchange with solvent deuterium

time-averages the two H**L1** environments, leading to a single signal set. Peaks at δ 6.86 (m) and 1.74 (s) of unknown origin were not removed during work-up.

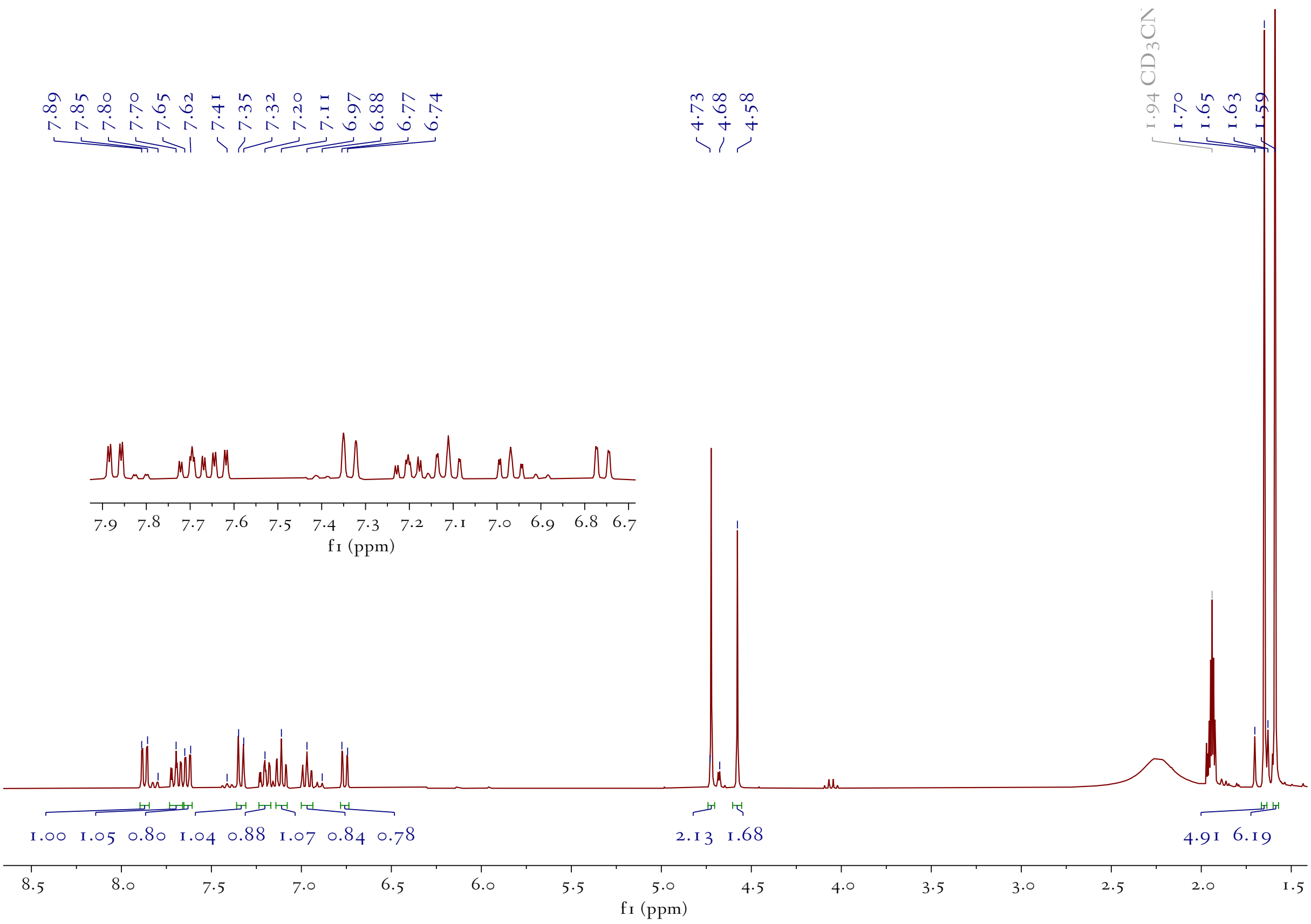

Fig. S11 Ion Pair Complex **1** in acetonitrile. ^{1}H NMR (300 MHz, CD_3CN): δ 10.54 (bs, 1H, OH), 7.87 (dd, *J* = 8.1, 1.7 Hz, 1H, H-6 Ar-A), 7.70 (ddd, *J* = 8.7, 7.1, 1.7 Hz, 1H, H-4 Ar-A), 7.63 (dd, *J* = 8.0, 1.8 Hz, ~1H, H-6 Ar-B), 7.34 (d, *J* = 8.5 Hz, 1H, H-3 Ar-A), 7.20 (ddd, *J* = 8.2, 7.3, 1.8 Hz, ~1H, H-4 Ar-B), 7.11 (m, 1H, H-5 Ar-A), 6.97 (m, ~1H, H-5 Ar-B), 6.75 (dd, *J* = 8.2, 0.9 Hz, ~1H, H-3 Ar-B), 4.68 (s, 2H, CH_2-A), 4.58 (s, ~2H, CH_2-B), 1.65 (s, ~5H, CH_3-B), 1.59 (s, 6H, CH_3-A). Minor signal set (~25% intensity) at: 7.80 (dd), 7.41 (ddd), 7.16 (ddd overlapping), 6.88 (d) in the aromatic region, attributed to a solution isomer or ion-pair conformer.

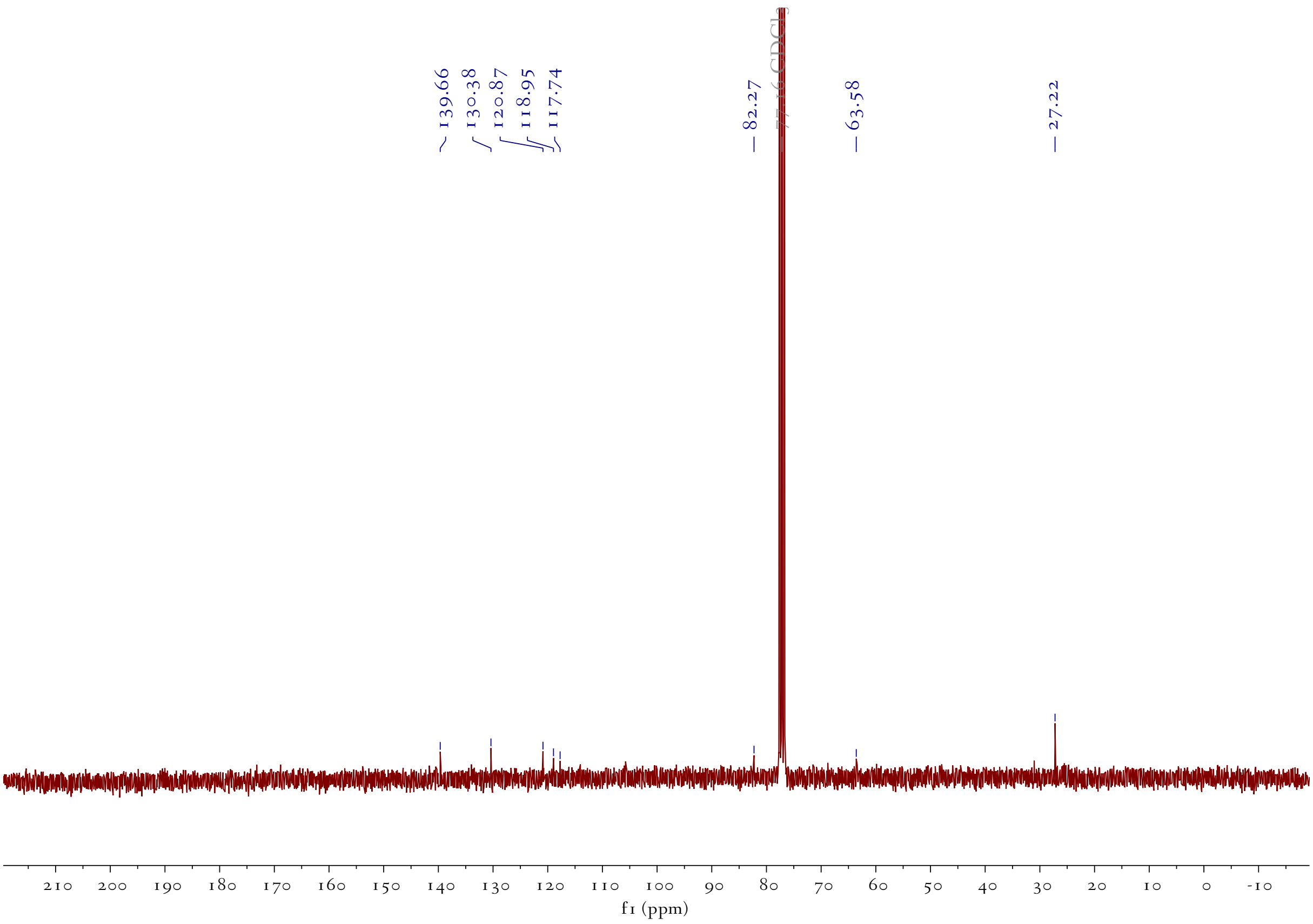

Fig. S12 ^{13}C NMR of **1** in $CDCl_3$. ^{13}C NMR (75 MHz, $CDCl_3$): δ 139.66, 130.38, 120.87, 118.95, 117.74, 82.27, 63.58, 27.77. Owing to the low solubility of **1** in $CDCl_3$, not all ^{13}C signals could be detected, even with an increased number of scans. A more complete spectrum was obtained in CD_3CN (**Fig. S13**).

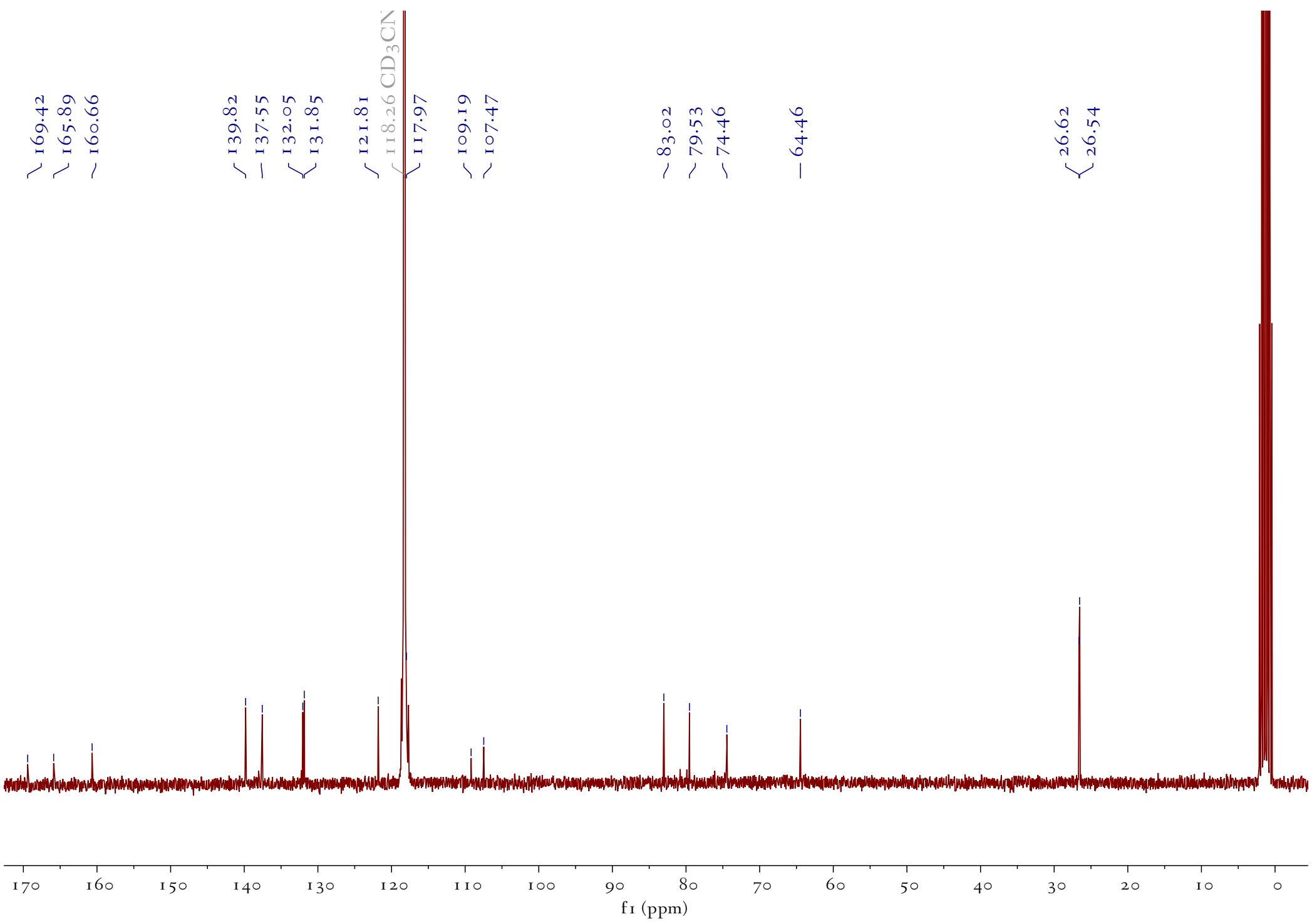

Fig. S13 ^{13}C NMR of complex **1** in acetonitrile. ^{13}C NMR (75 MHz, CD_3CN): δ 169.42, 165.89, 160.66, 139.82, 137.55, 132.05, 131.85, 121.81, 117.97, 109.19, 107.47, 83.02, 79.53, 74.46, 64.46, 26.62, 26.54.

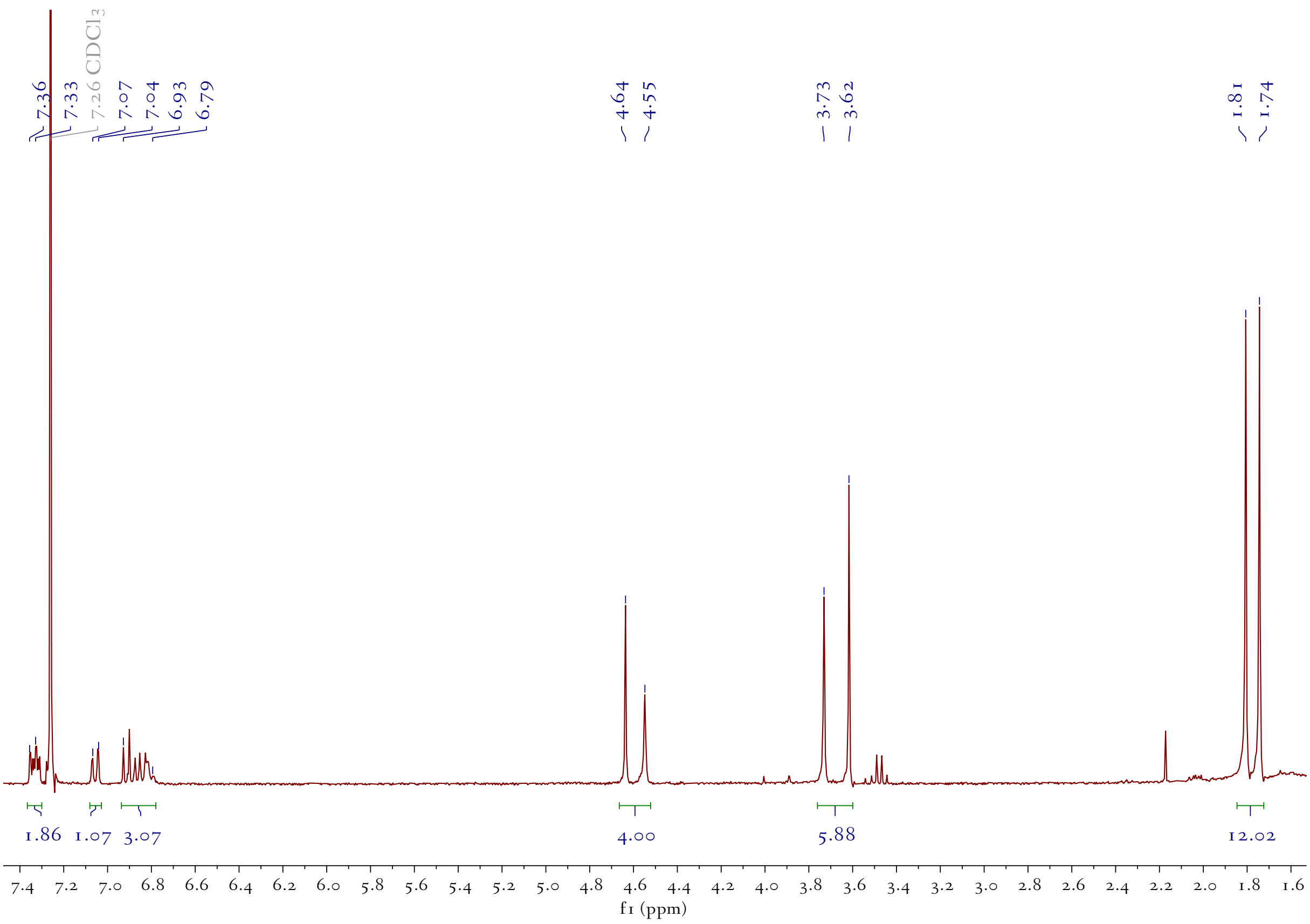

Fig. S14 ^{1}H NMR of Ion Pair Complex **2** (H_2dmozOMe)[ReOCl_3(dmozOMe)]. ^{1}H NMR (300 MHz, $CDCl_3$): δ 7.36–7.31 (dd, *J* = 8.3, 1.4 Hz, ~1H, Ar), 7.34–7.31 (dd, *J* = 7.6, 2.0 Hz, ~1H, Ar), 7.06 (dd, *J* = 8.3, 1.4 Hz, 1H, Ar), 6.93–6.79 (m, 3H, Ar), 4.64 (s, ~2H, CH_2), 4.55 (s, ~2H, CH_2), 3.73 (s, ~3H, OCH_3), 3.62 (s, ~3H, OCH_3), 1.81 (s, 6H, CH_3), 1.74 (s, 6H, CH_3). Unlike **1**, two distinct signal sets for both HL1 moieties are resolved in $CDCl_3$, consistent with stronger directional hydrogen bonding that slows the proton-exchange averaging process.

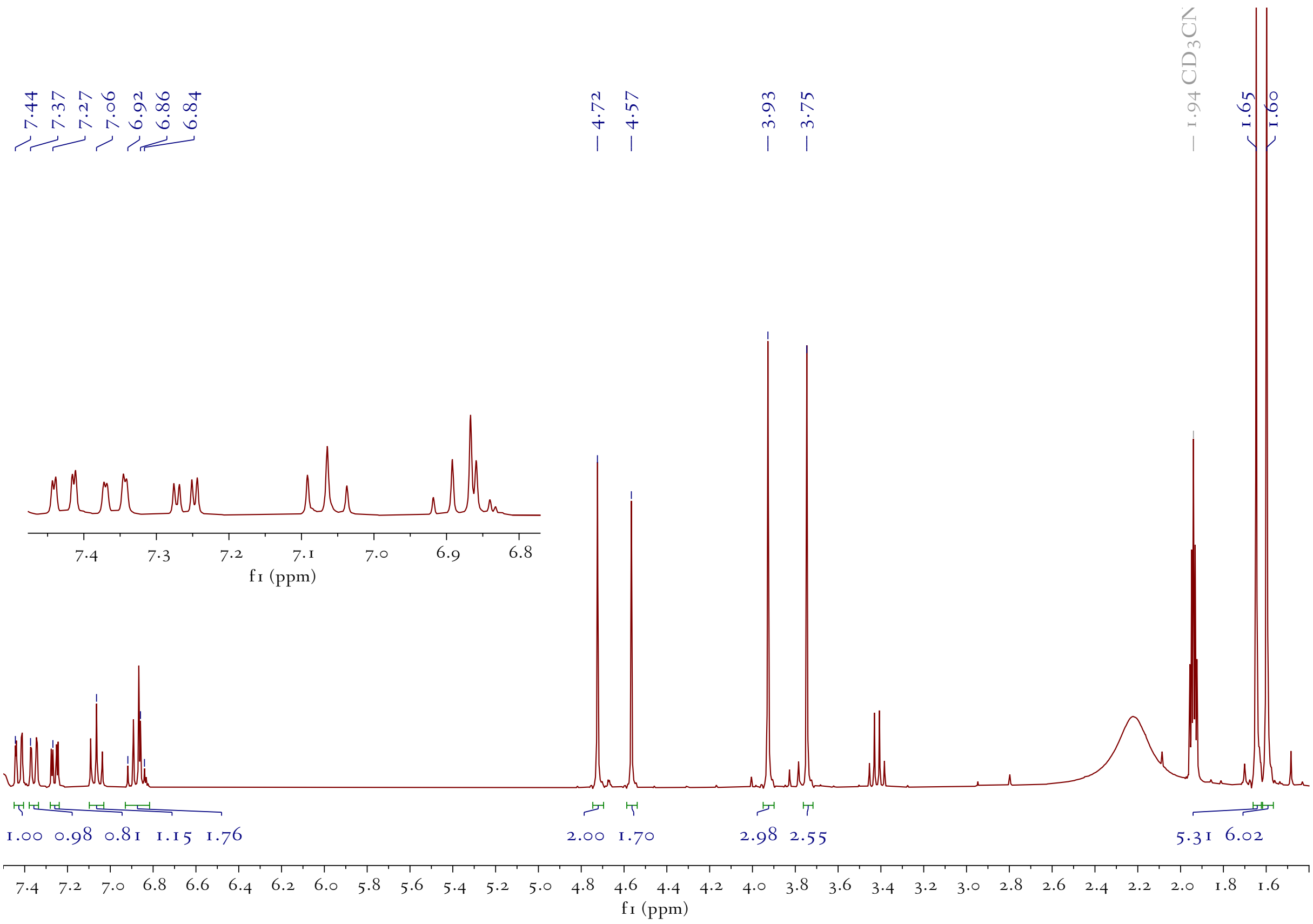

Fig. S15 ^{1}H NMR of Ion Pair Complex **2** in acetonitrile. ^{1}H NMR (300 MHz, CD_3CN): δ 7.43 (dd, *J* = 8.3, 1.4 Hz, 1H, Ar), 7.36 (dd, *J* = 8.2, 1.2 Hz, 1H, Ar), 7.26 (dd, *J* = 7.3, 2.2 Hz, ~1H, Ar), 7.06 (t, *J* = 8.2 Hz, ~1H, Ar), 6.92–6.84 (m, ~2H, Ar), 4.72 (s, 2H, CH_2), 4.57 (s, ~2H, CH_2), 3.93 (s, 3H, OCH_3), 3.75 (s, ~3H, OCH_3), 1.65 (s, ~6H, CH_3), 1.60 (s, 6H, CH_3).

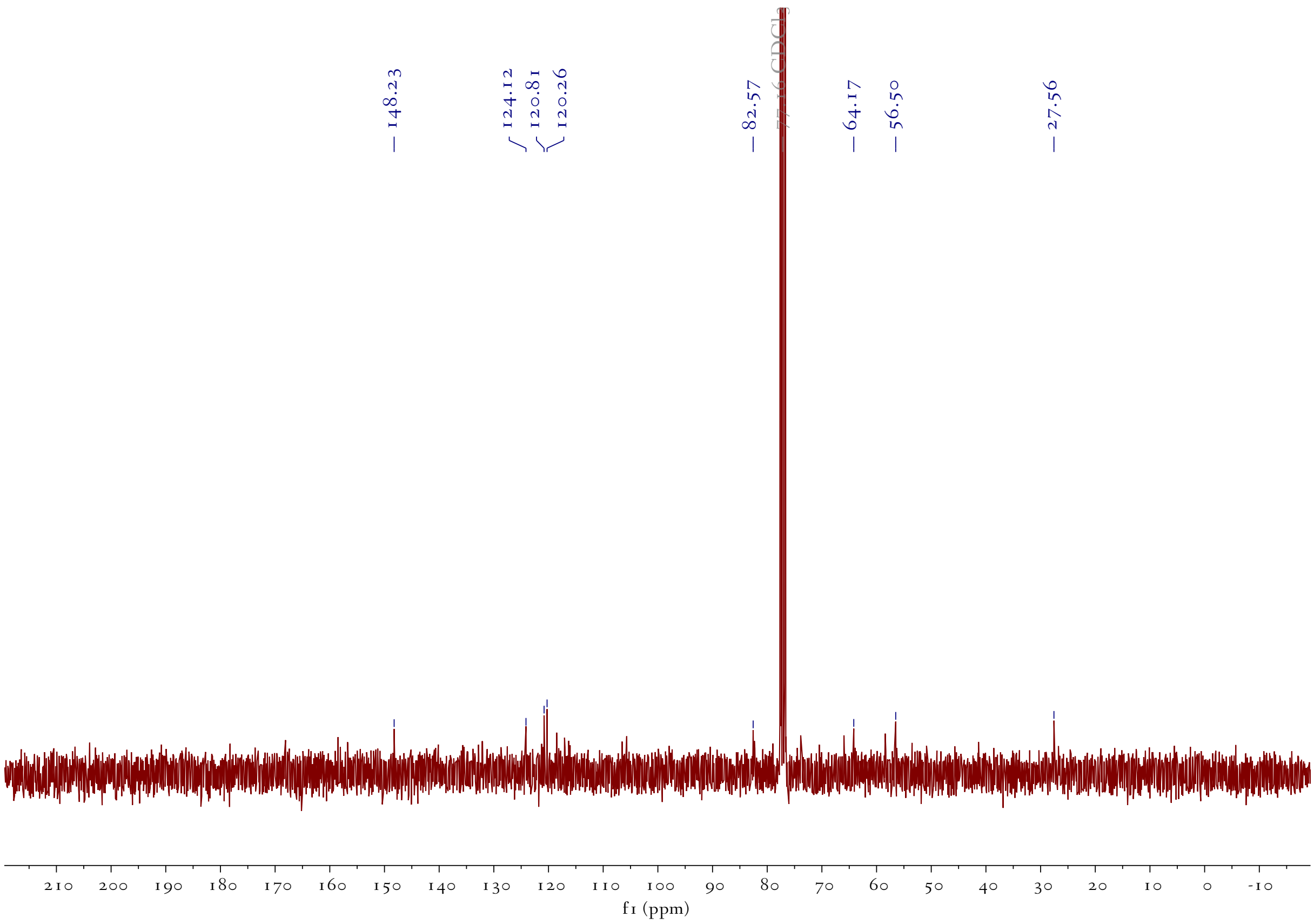

Fig. S16 ^{13}C NMR of complex 2 in $CDCl_3$. ^{13}C NMR (75 MHz, $CDCl_3$): δ 148.23, 124.12, 120.81, 120.26, 82.57, 65.50, 64.17, 27.56. Owing to the low solubility of **2**, not all ^{13}C signals could be detected, even with an increased number of scans.

Fully Coordinated Complex [ReOCl(dmoz)2] (3): Key NMR Diagnostic Features

The NMR spectra of **3** are readily distinguished from those of the ion pair complex **1** by three diagnostic features: (i) no significant difference between spectra in CD_3CN and $CDCl_3$; (ii) two fully resolved, inequivalent signal sets for the two bidentate HL1s; and (iii) geminal coupling of the $–CH_2–$ protons (two doublets per HL1, *J*gem ≈ 8–9 Hz), in contrast to the singlets observed for **1**. Similarly, two singlets are observed per methyl group (four total) in **3** due to the absence of the time-averaging process.

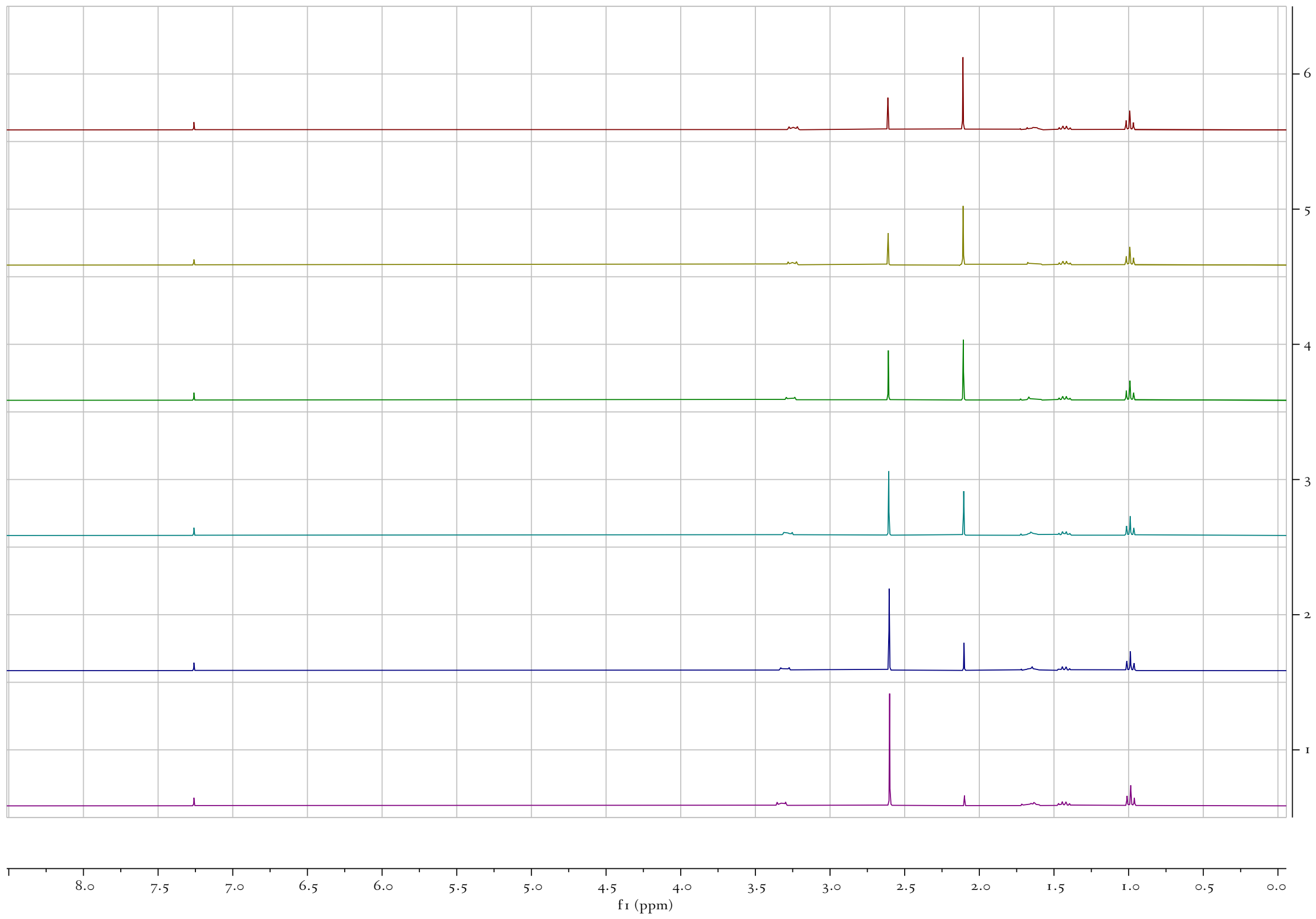

Fig. S17 Stacked ^{1}H NMR spectra (300 MHz, $CDCl_3$) of the catalytic perchlorate reduction by complex 1 (10 mol%, $[NBu_4][ClO_4]$, 4 equiv. DMS, N_2, rt) recorded over 25 h (top: start of the reaction; bottom: 1 h, 2 h, 3 h, 6 h, 10 h, 25 h). The conversion of DMS (δ 2.13) into DMSO (δ 2.55) is followed. No signals of the bis-chelate complex $[ReOCl(L1)_2]$ (3) are observed at any time, and the spectra of the catalytic mixture remain unchanged throughout the reaction.

Single Crystal X-Ray Diffraction Analysis

Complex 1. The X-ray data collection for complex **1** was performed with a Bruker AXS SMART APEXII CCD diffractometer at 100 K with Mo Kα radiation (λ= 0.71073 Å). Absorption corrections were made semi-empirically from equivalents. The structure was solved with the ShelXS-97[1] solution program using direct methods and refined with ShelXL 2014/6[2] using full matrix least squares minimization on F^2. The non-hydrogen atoms were refined with anisotropic displacement parameters without any constraints. The H atoms of the NH and OH groups were located on a difference Fourier map; the N–H distance was fixed at 0.88 Å and the O–H distance at 0.84 Å, with individual isotropic displacement parameters. Methylene H atoms were refined with common isotropic displacement parameters and idealized geometry (C–H = 0.99 Å). Aromatic C–H distances were set to 0.95 Å and methyl C–H distances to 0.98 Å.

Complex 2. The X-ray data collection for complex **2** was performed with a XtaLAB Synergy, Dualflex, HyPix-Arc 100 diffractometer at 100 K with Mo Kα radiation (λ= 0.71073 Å). Data reduction, scaling and absorption corrections were performed using the *CrysAlisPro* software.[2]A numerical absorption correction based on gaussian integration over a multifaceted crystal model and an empirical absorption correction using spherical harmonics, implemented in SCALE3 ABSPACK scaling algorithm, were performed. The structure was solved with the ShelXT 2018/2[3] solution program using the intrinsic phasing solution method and by using the Olex2[4] software as the graphical interface. The model was refined with ShelXL 2025/1[2] using full matrix least squares minimization on F^2. All non-hydrogen atoms were refined anisotropically. Hydrogen atom positions were calculated geometrically and refined using the riding model, except the NH and OH hydrogen atoms of the cation which were located on a difference Fourier map and refined freely.

CCDC 2574891 (complex **1**) and 2574892 (complex **2**) contain the supplementary crystallographic data for this paper. This data can be obtained free of charge via http://www.ccdc.cam.ac.uk/ or from Cambridge Crystallographic Data Centre, 12 Union Road, Cambridge, CB2 1EZ, UK.

Table S5 Crystal data and structure refinement for complexes **1** and **2**.

	1	2
Identification code	JSKK65	AD117
Empirical formula	$C_{11}H_{12}Cl_3NO_3Re \cdot C_{11}H_{14}NO_2$	$C_{12}H_{14}Cl_3NO_4Re \cdot C_{12}H_{16}NO_3$
Formula weight	691.00	751.05
Temperature /K	99.99	99.99(10)
Crystal system	monoclinic	orthorhombic
Space group	$P2_1/c$	$P2_12_12_1$
a /Å	10.6059(5)	9.1936(2)
b /Å	12.2574(6)	16.7648(3)
c / Å	19.0613(9)	17.9504(3)
α /°	90	90
β /°	99.844(2)	90
γ /°	90	90
Volume /$Å^3$	2441.5(2)	2766.67(9)
Z	4	4
$ρ_{calc}$ g/cm^3	1.880	1.803
μ /mm^{-1}	5.342	4.727
F(000)	1352.0	1480.0
Crystal size /mm^3	0.27 × 0.26 × 0.09	0.15 × 0.12 × 0.09
Radiation	MoKα (λ = 0.71073 Å)	Mo Kα (λ = 0.71073 Å)
2Θ range for data collection /°	3.96 to 80	4.978 to 61.01
Index ranges	-19 ≤ h ≤ 19,<br>-22 ≤ k ≤ 13,<br>-34 ≤ l ≤ 34	-12 ≤ h ≤ 13,<br>-23 ≤ k ≤ 23,<br>-25 ≤ l ≤ 25
Reflections collected	56875	42675
Independent reflections	15131<br>[R_{int} = 0.0419, R_{sigma} = 0.0387]	8433<br>[R_{int} = 0.0616, R_{sigma} = 0.0446]
Data/restraints/ parameters	15131 / 2 / 318	8433 / 0 / 348
Goodness-of-fit on F^2	1.051	1.010
Final R indexes [I≥ 2σ(I)]	R_1 = 0.0256, wR_2 = 0.0524	R_1 = 0.0286, wR_2 = 0.0574
Final R indexes [all data]	R_1 = 0.0349, wR_2 = 0.0557	R_1 = 0.0325, wR_2 = 0.0584
Largest diff. peak/hole / e.$Å^{-3}$	2.00 / -1.83	1.37 / -1.00

Flack Parameter		-0.019(4)

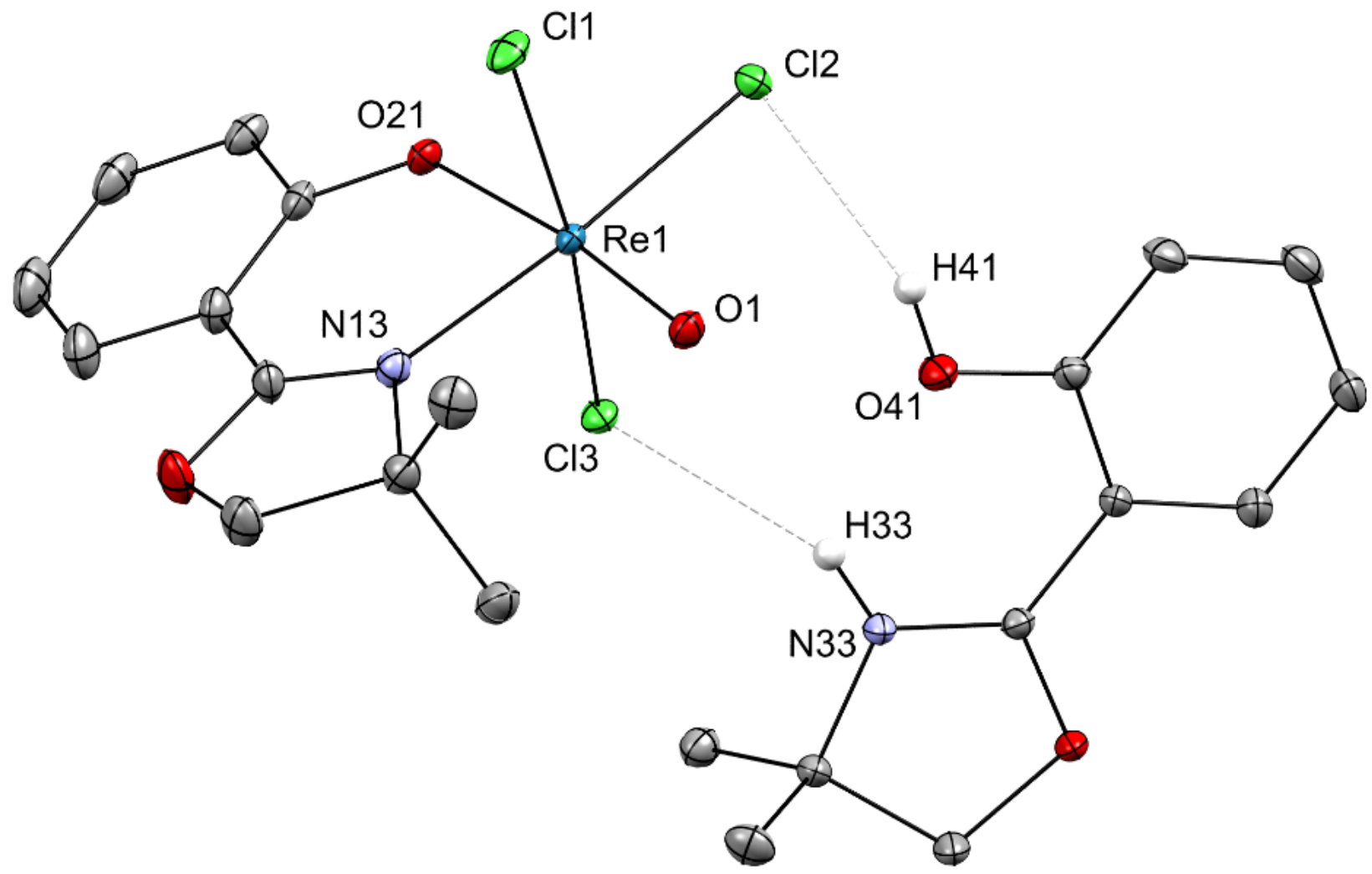

Fig. S18 Molecular structure of complex **1** with ellipsoids at 50% probability. Hydrogen atoms are omitted for clarity, except those of the OH and NH groups. Hydrogen Bonds are depicted as dashed lines.

Table S6 Selected bond lengths (Å), angles (°) and hydrogen bonding information for complex **1**.

Bond	Length /Å	Bond	Length /Å
Re1–O1	1.6826(11)	Re1–Cl1	2.3714(4)
Re1–O21	1.9651(11)	Re1–Cl2	2.4028(4)
Re1–N13	2.1215(14)	Re1–Cl3	2.4268(4)
C12–N13	1.295(2)	N13–C14	1.509(2)
O21–C21	1.3326(19)	O41–C41	1.357(2)
C32–N33	1.299(2)	N33–C34	1.4846(19)

Angle	Value /°	Angle	Value /°
O1–Re1–O21	172.90(5)	N13–Re1–Cl2	173.47(4)
Cl1–Re1–Cl3	170.182(14)	C12–N13–Re1	126.72(11)
C12–N13–C14	108.81(14)	C14–N13–Re1	124.04(10)
C21–O21–Re1	136.15(10)		

D	H	A	d(D-H)/Å	d(H-A)/Å	d(D-A)/Å	D-H-A/deg
O41	H41	Cl21	0.84	2.403(16)	3.1720(12)	153(3)
N33	H33	Cl11	0.88	2.130(17)	2.6803(17)	119.9(15)
N33	H33	O41	0.88	2.466(13)	3.2414(13)	147(2)

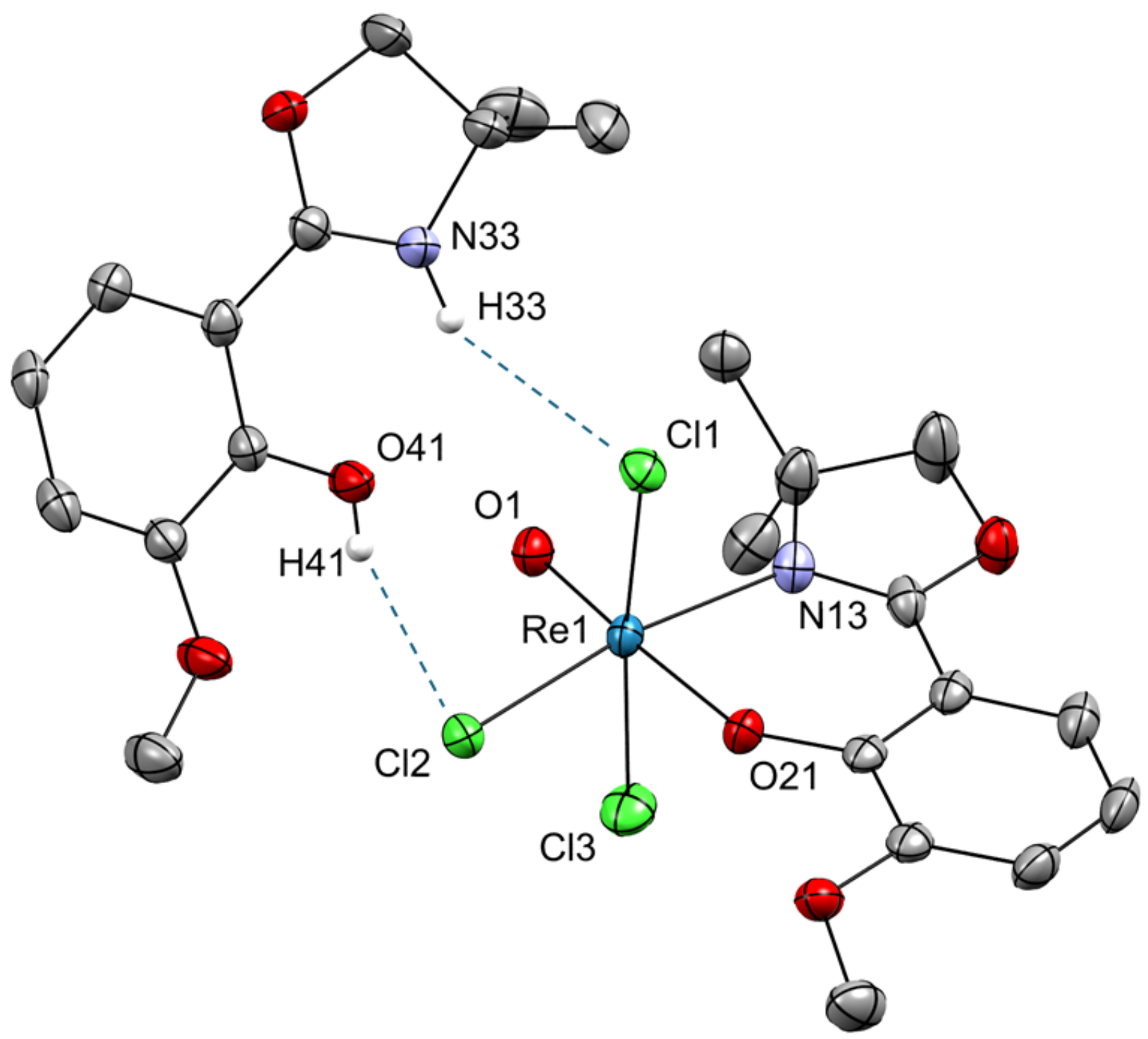

Fig. S19 Molecular structure of complex **2** with ellipsoids at 50% probability. Hydrogen atoms are omitted for clarity, except those of the OH and NH groups. Hydrogen Bonds are depicted as dashed lines.

Table S7 Selected bond lengths (Å), angles (°) and hydrogen bonding information for complex **2**.

Bond	Length /Å	Bond	Length /Å
Re1–O1	1.684(3)	Re1–Cl1	2.4048(11)
Re1–O21	1.973(3)	Re1–Cl2	2.3899(11)
Re1–N13	2.133(4)	Re1–Cl3	2.3918(12)
C12–N13	1.292(6)	N13–C14	1.517(6)
O21–C21	1.326(5)	O41–C41	1.344(5)
C32–N33	1.297(6)	N33–C34	1.488(6)

Angle	Value /°	Angle	Value /°
O1–Re1–O21	175.80(14)	N13–Re1–Cl2	168.74(11)
Cl1–Re1–Cl3	173.57(5)	C12–N13–Re1	127.7(3)
C12–N13–C14	108.8(4)	C14–N13–Re1	123.3(3)
C21–O21–Re1	139.4(3)		

D	H	A	d(D-H)/Å	d(H-A)/Å	d(D-A)/Å	D-H-A/deg
O41	H41	Cl21	0.71(6)	2.42(6)	3.070(4)	151(6)
N33	H33	Cl11	0.86(5)	2.63(5)	3.407(4)	151(4)
N33	H33	O41	0.86(5)	2.05(5)	2.614(5)	122(4)

DFT Data

All DFT calculations were performed with TURBOMOLE 7.9.[5–8] Geometries were optimized using the PBE[9] density functional approximations, together with the D3BJ dispersion correction[10,11] and the dhf-SVP basis set.[12] Convergence criteria for the geometries were set to at least 10⁻⁷ Hartree and 10⁻⁴ Hartree/bohr. The SCF was converged to 10⁻⁹ Hartree and the m5 integration grid was used.

Solvent effects were included by employing the COSMO solvation model for the solvents discussed in the main text.[13] To account for relativistic effects at the rhenium center, the corresponding dhf-ECP (effective core potential) was used.[14] To accelerate the calculations, the resolution-of-identity approximation (RI-J) was applied.[15–18]

Analytical normal modes were determined using TURBOMOLE's *aoforce* program to confirm the stationary points. The rigid-rotor–harmonic-oscillator (RRHO) approximation was used to calculate zero-point vibrational energies and thermal properties (298.15 and 333.15 K, 1 bar).

Subsequently, wB97X-D3[19]/dhf-TZVPP[12] and B3LYP[20–22]-D3BJ/dhf-TZVPP single-point calculations were performed at the respective dhf-SVP geometries, again using the RI-J approximation and the COSMO solvation model as well as seminumerical exchange (senex, grid m5).[23–25] Zero-point energies and thermal corrections for the single-point calculations were taken from the dhf-SVP results. The data reported in the main text are the wB97X-D3/dhf-TZVPP(COSMO) single-point results, unless otherwise noted.

Table S8 Solvent dependence of computed Gibbs free energies of association (ΔG°, kJ mol^{-1}) for contact ion-pair complex **1** and bis-chelate isomer **3**. B3LYP/dhf-TZVPP//PBE-D3BJ/dhf-SVP, COSMO (25 °C, 1 bar).

**Solvent**	**ε_r**	**ΔG CIP (1) / kJ mol^{-1}**	**ΔG bis-chelate (3) / kJ mol^{-1}**	**ΔG (1) vs (3) / kJ mol^{-1}**
Acetonitrile	37.5	+4.0	+29.5	−25.5
Methanol	32.6	+2.0	+27.1	−25.1
Ethanol	24.3	−3.0	+21.1	−24.1
Acetone	20.7	−6.7	+16.9	−23.7
Dichloromethane	8.93	−36.9	−17.9	−19.0
Ethyl acetate	6.02	−61.6	−45.9	−15.6
Chloroform	4.81	−79.3	−65.8	−13.5
Toluene	2.38	−160.4	−155.4	−5.1

Table S9 Solvent dependence of computed Gibbs free energies of association (ΔG°, kJ mol^{-1}) for contact ion-pair complex **2** and bis-chelate isomer **4**. B3LYP/dhf-TZVPP//PBE-D3BJ/dhf-SVP, COSMO (25 °C, 1 bar).

**Solvent**	**ε_r**	**ΔG CIP (2) / kJ mol^{-1}**	**ΔG bis-chelate (4) / kJ mol^{-1}**	**ΔG (2) vs (4) / kJ mol^{-1}**
Acetonitrile	37.5	+2.5	+31.2	−28.7
Methanol	32.6	−0.1	+28.9	−29.0
Ethanol	24.3	−4.2	+23.0	−27.2
Acetone	20.7	−7.2	+19.1	−26.3
Dichloromethane	8.93	−35.2	−14.6	−20.6
Ethyl acetate	6.02	−59.7	−41.3	−18.4
Chloroform	4.81	−74.2	−60.3	−13.9
Toluene	2.38	−150.2	−146.5	−3.7

Table S10 Solvent dependence of computed Gibbs free energies of association (ΔG°, kJ mol^{-1}) for contact ion-pair complex **1** and bis-chelate isomer **3**. ωB97X-D3/dhf-TZVPP//PBE-D3BJ/dhf-SVP, COSMO (60 °C, 1 bar).

Solvent	ε_r	ΔG CIP (1) / kJ mol^{-1}	ΔG bis-chelate (3) / kJ mol^{-1}	ΔG (1) vs (3) / kJ mol^{-1}
Acetonitrile	37.5	+17.2	+30.9	−13.7
Methanol	32.6	+15.3	+28.5	−13.3
Ethanol	24.3	+10.3	+22.5	−12.2
Acetone	20.7	+6.7	+18.4	−11.7
Dichloromethane	8.93	−23.1	−16.5	−6.5
Ethyl acetate	6.02	−47.4	−44.7	−2.8
Chloroform	4.81	−65.0	−64.7	−0.3
Toluene	2.38	−145.6	−154.9	+9.3

Table S11 Solvent dependence of computed Gibbs free energies of association (ΔG°, kJ mol^{-1}) for contact ion-pair complex **2** and bis-chelate isomer **4**. ωB97X-D3/dhf-TZVPP//PBE-D3BJ/dhf-SVP, COSMO (60 °C, 1 bar).

Solvent	ε_r	ΔG CIP (2) / kJ mol^{-1}	ΔG bis-chelate (4) / kJ mol^{-1}	ΔG (2) vs (4) / kJ mol^{-1}
Acetonitrile	37.5	+13.4	+32.2	−18.8
Methanol	32.6	+10.7	+29.9	−19.2
Ethanol	24.3	+6.8	+24.0	−17.3
Acetone	20.7	+3.9	+20.1	−16.3
Dichloromethane	8.93	−23.6	−13.4	−10.2
Ethyl acetate	6.02	−47.9	−40.2	−7.8
Chloroform	4.81	−62.1	−59.2	−2.9
Toluene	2.38	−137.3	−145.9	+8.6

Table S12 Energies calculated with different DFT methods.

PBE+D3BJ/dhf-SVP — RT (25 °C), 1 bar		
**Acetone**		
Species	E (Eh)	chem. pot. (kJ mol^{-1})
**1**	-2795.689891	987.30
**2**	-3024.318391	1126.51
**3**	-1874.700272	946.52
**4**	-2103.333750	1095.03
HCl	-460.466254	-29.99
**HL1**	-631.479073	498.51
**HL2**	-745.797101	572.63
Re**L1**	-2164.175778	432.42
Re**L2**	-2278.491119	507.05
**MeCN**		
Species	E (Eh)	chem. pot. (kJ mol^{-1})
**1**	-2795.691669	986.96
**2**	-3024.320713	1126.53
**3**	-1874.701253	946.40
**4**	-2103.335066	1094.97
HCl	-460.466433	-30.00
**HL1**	-631.481647	498.39
**HL2**	-745.799479	572.52
Re**L1**	-2164.179243	432.42
Re**L2**	-2278.494938	507.09
**$CHCl_3$**		
Species	E (Eh)	chem. pot. (kJ mol^{-1})
**1**	-2795.679048	989.34
**2**	-3024.304487	1126.33
**3**	-1874.694182	947.14
**4**	-2103.325633	1095.92
HCl	-460.465092	-29.91
**HL1**	-631.461846	499.26
**HL2**	-745.781146	573.32
Re**L1**	-2164.153604	432.41
Re**L2**	-2278.466808	506.86
**DCM**		
Species	E (Eh)	chem. pot. (kJ mol^{-1})
**1**	-2795.685130	988.32
**2**	-3024.312231	1126.20
**3**	-1874.697620	946.82
**4**	-2103.330209	1095.26
HCl	-460.465760	-29.95
**HL1**	-631.471849	498.81
**HL2**	-745.790417	572.86

Re**L1**	-2164.166275	432.44
Re**L2**	-2278.480673	506.95

Toluene		
Species	E (Eh)	chem. pot. (kJ mol^{-1})
**1**	-2795.668793	991.13
**2**	-3024.291699	1127.09
**3**	-1874.688237	947.82
**4**	-2103.317808	1096.66
HCl	-460.463875	-29.84
**HL1**	-631.442790	499.99
**HL2**	-745.763437	574.08
Re**L1**	-2164.130749	432.43
Re**L2**	-2278.442013	506.34

MeOH		
Species	E (Eh)	chem. pot. (kJ mol^{-1})
**1**	-2795.691333	987.04
**2**	-3024.320257	1125.65
**3**	-1874.701067	946.41
**4**	-2103.334818	1094.97
HCl	-460.466399	-30.00
**HL1**	-631.481166	498.41
**HL2**	-745.799035	572.54
Re**L1**	-2164.178591	432.40
Re**L2**	-2278.494219	507.08

EtOH		
Species	E (Eh)	chem. pot. (kJ mol^{-1})
**1**	-2795.690465	987.30
**2**	-3024.319139	1126.34
**3**	-1874.700590	946.48
**4**	-2103.334181	1095.03
HCl	-460.466313	-29.99
**HL1**	-631.479918	498.47
**HL2**	-745.797881	572.59
Re**L1**	-2164.176910	432.42
Re**L2**	-2278.492366	507.09

EtOAc		
Species	E (Eh)	chem. pot. (kJ mol⁻^{1})
**1**	-2795.681523	989.00
**2**	-3024.307614	1124.19
**3**	-1874.695588	947.05
**4**	-2103.327501	1095.62
HCl	-460.465369	-29.93
**HL1**	-631.466025	499.13
**HL2**	-745.785022	573.12

ReL1	-2164.158834	432.43
ReL2	-2278.472523	506.87

B3LYP+D3BJ/dhf-TZVPP//PBE+D3BJ/dhf-SVP — RT (25 °C), 1 bar		
Acetone		
Species	E (Eh)	chem. pot. (kJ mol^{-1})
**1**	-2798.860847	987.30
**2**	-3027.893210	1126.51
**3**	-1877.268883	946.52
**4**	-2106.303789	1095.03
HCl	-460.772284	-29.99
**HL1**	-632.593678	498.51
**HL2**	-747.112525	572.63
Re**L1**	-2166.243131	432.42
Re**L2**	-2280.760089	507.05
MeCN		
Species	E (Eh)	chem. pot. (kJ mol^{-1})
**1**	-2798.862862	986.96
**2**	-3027.895904	1126.53
**3**	-1877.269947	946.40
**4**	-2106.305185	1094.97
HCl	-460.772459	-30.00
**HL1**	-632.596286	498.39
**HL2**	-747.114960	572.52
Re**L1**	-2166.246711	432.42
Re**L2**	-2280.764012	507.09
$CHCl_3$		
Species	E (Eh)	chem. pot. (kJ mol^{-1})
**1**	-2798.848612	989.34
**2**	-3027.877176	1126.33
**3**	-1877.262318	947.14
**4**	-2106.295214	1095.92
HCl	-460.771156	-29.91
**HL1**	-632.576215	499.26
**HL2**	-747.096198	573.32
Re**L1**	-2166.220240	432.41
Re**L2**	-2280.735132	506.86
DCM		
Species	E (Eh)	chem. pot. (kJ mol^{-1})
**1**	-2798.855474	988.32
**2**	-3027.886102	1126.20
**3**	-1877.266005	946.82
**4**	-2106.300050	1095.26

HCl	-460.771803	-29.95
**HL1**	-632.586358	498.81
**HL2**	-747.105674	572.86
Re**L1**	-2166.233318	432.44
Re**L2**	-2280.749364	506.95

Toluene		
Species	E (Eh)	chem. pot. (kJ mol^{-1})
**1**	-2798.837035	991.13
**2**	-3027.862709	1127.09
**3**	-1877.255910	947.82
**4**	-2106.287017	1096.66
HCl	-460.769981	-29.84
**HL1**	-632.556905	499.99
**HL2**	-747.078069	574.08
Re**L1**	-2166.196660	432.43
Re**L2**	-2280.709647	506.34

MeOH		
Species	E (Eh)	chem. pot. (kJ mol^{-1})
**1**	-2798.862479	987.04
**2**	-3027.895357	1125.65
**3**	-1877.269754	946.41
**4**	-2106.304922	1094.97
HCl	-460.772426	-30.00
**HL1**	-632.595798	498.41
**HL2**	-747.114504	572.54
Re**L1**	-2166.246035	432.40
Re**L2**	-2280.763274	507.08

EtOH		
Species	E (Eh)	chem. pot. (kJ mol^{-1})
**1**	-2798.861475	987.30
**2**	-3027.894080	1126.34
**3**	-1877.269225	946.48
**4**	-2106.304257	1095.03
HCl	-460.772341	-29.99
**HL1**	-632.594533	498.47
**HL2**	-747.113325	572.59
Re**L1**	-2166.244309	432.42
Re**L2**	-2280.761366	507.09

EtOAc		
Species	E (Eh)	chem. pot. (kJ mol^{-1})
**1**	-2798.851395	989.00
**2**	-3027.880724	1124.19
**3**	-1877.263814	947.05
**4**	-2106.297189	1095.62

HCl	-460.771424	-29.93
**HL1**	-632.580441	499.13
**HL2**	-747.100157	573.12
Re**L1**	-2166.225627	432.43
Re**L2**	-2280.740996	506.87

ωB97X-D3/dhf-TZVPP//PBE+D3BJ/dhf-SVP — RT (25 °C), 1 bar		
Acetone		
Species	E (Eh)	chem. pot. (kJ mol^{-1})
**1**	-2799.332976	987.30
**2**	-3028.408180	1126.51
**3**	-1877.641046	946.52
**4**	-2106.718423	1095.03
HCl	-460.822877	-29.99
**HL1**	-632.739350	498.51
**HL2**	-747.279433	572.63
Re**L1**	-2166.572344	432.42
Re**L2**	-2281.110412	507.05
MeCN		
Species	E (Eh)	chem. pot. (kJ mol^{-1})
**1**	-2799.335134	986.96
**2**	-3028.411012	1126.53
**3**	-1877.642178	946.40
**4**	-2106.719895	1094.97
HCl	-460.823057	-30.00
**HL1**	-632.741959	498.39
**HL2**	-747.281867	572.52
Re**L1**	-2166.576003	432.42
Re**L2**	-2281.114401	507.09
$CHCl_3$		
Species	E (Eh)	chem. pot. (kJ mol^{-1})
**1**	-2799.319839	989.34
**2**	-3028.391208	1126.33
**3**	-1877.634031	947.14
**4**	-2106.709361	1095.92
HCl	-460.821716	-29.91
**HL1**	-632.721883	499.26
**HL2**	-747.263112	573.32
Re**L1**	-2166.548848	432.41
Re**L2**	-2281.084903	506.86
DCM		
Species	E (Eh)	chem. pot. (kJ mol^{-1})
**1**	-2799.327227	988.32
**2**	-3028.400676	1126.20

**3**	-1877.637967	946.82
**4**	-2106.714475	1095.26
HCl	-460.822382	-29.95
**HL1**	-632.732029	498.81
**HL2**	-747.272586	572.86
Re**L1**	-2166.562290	432.44
Re**L2**	-2281.099473	506.95

Toluene		
**Species**	**E (Eh)**	**chem. pot. (kJ mol^{-1})**
**1**	-2799.307346	991.13
**2**	-3028.375752	1127.09
**3**	-1877.627157	947.82
**4**	-2106.700704	1096.66
HCl	-460.820507	-29.84
**HL1**	-632.702567	499.99
**HL2**	-747.244983	574.08
Re**L1**	-2166.524508	432.43
Re**L2**	-2281.058676	506.34

MeOH		
**Species**	**E (Eh)**	**chem. pot. (kJ mol^{-1})**
**1**	-2799.334724	987.04
**2**	-3028.410446	1125.65
**3**	-1877.641973	946.41
**4**	-2106.719619	1094.97
HCl	-460.823023	-30.00
**HL1**	-632.741471	498.41
**HL2**	-747.281411	572.54
Re**L1**	-2166.575311	432.40
Re**L2**	-2281.113652	507.08

EtOH		
**Species**	**E (Eh)**	**chem. pot. (kJ mol^{-1})**
**1**	-2799.333652	987.30
**2**	-3028.409097	1126.34
**3**	-1877.641408	946.48
**4**	-2106.718921	1095.03
HCl	-460.822936	-29.99
**HL1**	-632.740206	498.47
**HL2**	-747.280232	572.59
Re**L1**	-2166.573549	432.42
Re**L2**	-2281.111708	507.09

EtOAc		
**Species**	**E (Eh)**	**chem. pot. (kJ mol^{-1})**
**1**	-2799.322832	989.00
**2**	-3028.394954	1124.19

3	-1877.635622	947.05
4	-2106.711456	1095.62
HCl	-460.821992	-29.93
HL1	-632.726110	499.13
HL2	-747.267069	573.12
ReL1	-2166.554386	432.43
ReL2	-2281.090914	506.87

PBE+D3BJ/dhf-SVP — 60 °C, 1 bar		
Acetone		
Species	E (Eh)	chem. pot. (kJ mol^{-1})
1	-2795.689891	954.08
2	-3024.318391	1088.55
3	-1874.700272	917.75
4	-2103.333750	1062.50
HCl	-460.466254	-36.60
HL1	-631.479073	481.53
HL2	-745.797101	553.75
ReL1	-2164.175778	410.02
ReL2	-2278.491119	482.81
MeCN		
Species	E (Eh)	chem. pot. (kJ mol^{-1})
1	-2795.691669	953.70
2	-3024.320713	1088.57
3	-1874.701253	917.63
4	-2103.335066	1062.45
HCl	-460.466433	-36.61
HL1	-631.481647	481.40
HL2	-745.799479	553.65
ReL1	-2164.179243	410.02
ReL2	-2278.494938	482.84
$CHCl_3$		
Species	E (Eh)	chem. pot. (kJ mol^{-1})
1	-2795.679048	956.33
2	-3024.304487	1088.40
3	-1874.694182	918.39
4	-2103.325633	1063.44
HCl	-460.465092	-36.52
HL1	-631.461846	482.32
HL2	-745.781146	554.46
ReL1	-2164.153604	410.03
ReL2	-2278.466808	482.62
DCM		
Species	E (Eh)	chem. pot. (kJ mol^{-1})
1	-2795.685130	955.21

**2**	-3024.312231	1088.22
**3**	-1874.697620	918.06
**4**	-2103.330209	1062.73
HCl	-460.465760	-36.57
**HL1**	-631.471849	481.84
**HL2**	-745.790417	553.98
Re**L1**	-2164.166275	410.05
Re**L2**	-2278.480673	482.70

Toluene		
Species	E (Eh)	chem. pot. (kJ mol^{-1})
**1**	-2795.668793	958.33
**2**	-3024.291699	1089.26
**3**	-1874.688237	919.09
**4**	-2103.317808	1064.20
HCl	-460.463875	-36.45
**HL1**	-631.442790	483.08
**HL2**	-745.763437	555.25
Re**L1**	-2164.130749	410.08
Re**L2**	-2278.442013	482.07

MeOH		
Species	E (Eh)	chem. pot. (kJ mol^{-1})
**1**	-2795.691333	953.79
**2**	-3024.320257	1087.59
**3**	-1874.701067	917.64
**4**	-2103.334818	1062.44
HCl	-460.466399	-36.61
**HL1**	-631.481166	481.42
**HL2**	-745.799035	553.67
Re**L1**	-2164.178591	410.00
Re**L2**	-2278.494219	482.83

EtOH		
Species	E (Eh)	chem. pot. (kJ mol^{-1})
**1**	-2795.690465	954.08
**2**	-3024.319139	1088.37
**3**	-1874.700590	917.72
**4**	-2103.334181	1062.50
HCl	-460.466313	-36.60
**HL1**	-631.479918	481.49
**HL2**	-745.797881	553.72
Re**L1**	-2164.176910	410.02
Re**L2**	-2278.492366	482.84

EtOAc		
Species	E (Eh)	chem. pot. (kJ mol^{-1})
**1**	-2795.681523	955.96

**2**	-3024.307614	1086.00
**3**	-1874.695588	918.30
**4**	-2103.327501	1063.12
HCl	-460.465369	-36.54
**HL1**	-631.466025	482.19
**HL2**	-745.785022	554.26
Re**L1**	-2164.158834	410.04
Re**L2**	-2278.472523	482.62

B3LYP+D3BJ/dhf-TZVPP//PBE+D3BJ/dhf-SVP — 60 °C, 1 bar		
Acetone		
Species	E (Eh)	chem. pot. (kJ mol^{-1})
**1**	-2798.860847	954.08
**2**	-3027.893210	1088.55
**3**	-1877.268883	917.75
**4**	-2106.303789	1062.50
HCl	-460.772284	-36.60
**HL1**	-632.593678	481.53
**HL2**	-747.112525	553.75
Re**L1**	-2166.243131	410.02
Re**L2**	-2280.760089	482.81
MeCN		
Species	E (Eh)	chem. pot. (kJ mol^{-1})
**1**	-2798.862862	953.70
**2**	-3027.895904	1088.57
**3**	-1877.269947	917.63
**4**	-2106.305185	1062.45
HCl	-460.772459	-36.61
**HL1**	-632.596286	481.40
**HL2**	-747.114960	553.65
Re**L1**	-2166.246711	410.02
Re**L2**	-2280.764012	482.84
$CHCl_3$		
Species	E (Eh)	chem. pot. (kJ mol^{-1})
**1**	-2798.848612	956.33
**2**	-3027.877176	1088.40
**3**	-1877.262318	918.39
**4**	-2106.295214	1063.44
HCl	-460.771156	-36.52
**HL1**	-632.576215	482.32
**HL2**	-747.096198	554.46
Re**L1**	-2166.220240	410.03
Re**L2**	-2280.735132	482.62
DCM		

Species	E (Eh)	chem. pot. (kJ mol^{-1})
**1**	-2798.855474	955.21
**2**	-3027.886102	1088.22
**3**	-1877.266005	918.06
**4**	-2106.300050	1062.73
HCl	-460.771803	-36.57
**HL1**	-632.586358	481.84
**HL2**	-747.105674	553.98
Re**L1**	-2166.233318	410.05
Re**L2**	-2280.749364	482.70

Toluene		
Species	E (Eh)	chem. pot. (kJ mol^{-1})
**1**	-2798.837035	958.33
**2**	-3027.862709	1089.26
**3**	-1877.255910	919.09
**4**	-2106.287017	1064.20
HCl	-460.769981	-36.45
**HL1**	-632.556905	483.08
**HL2**	-747.078069	555.25
Re**L1**	-2166.196660	410.08
Re**L2**	-2280.709647	482.07

MeOH		
Species	E (Eh)	chem. pot. (kJ mol^{-1})
**1**	-2798.862479	953.79
**2**	-3027.895357	1087.59
**3**	-1877.269754	917.64
**4**	-2106.304922	1062.44
HCl	-460.772426	-36.61
**HL1**	-632.595798	481.42
**HL2**	-747.114504	553.67
Re**L1**	-2166.246035	410.00
Re**L2**	-2280.763274	482.83

EtOH		
Species	E (Eh)	chem. pot. (kJ mol^{-1})
**1**	-2798.861475	954.08
**2**	-3027.894080	1088.37
**3**	-1877.269225	917.72
**4**	-2106.304257	1062.50
HCl	-460.772341	-36.60
**HL1**	-632.594533	481.49
**HL2**	-747.113325	553.72
Re**L1**	-2166.244309	410.02
Re**L2**	-2280.761366	482.84

EtOAc

Species	E (Eh)	chem. pot. (kJ mol^{-1})
**1**	-2798.851395	955.96
**2**	-3027.880724	1086.00
**3**	-1877.263814	918.30
**4**	-2106.297189	1063.12
HCl	-460.771424	-36.54
**HL1**	-632.580441	482.19
**HL2**	-747.100157	554.26
Re**L1**	-2166.225627	410.04
Re**L2**	-2280.740996	482.62

ωB97X-D3/dhf-TZVPP//PBE+D3BJ/dhf-SVP — 60 °C, 1 bar		
Acetone		
Species	E (Eh)	chem. pot. (kJ mol^{-1})
**1**	-2799.332976	954.08
**2**	-3028.408180	1088.55
**3**	-1877.641046	917.75
**4**	-2106.718423	1062.50
HCl	-460.822877	-36.60
**HL1**	-632.739350	481.53
**HL2**	-747.279433	553.75
Re**L1**	-2166.572344	410.02
Re**L2**	-2281.110412	482.81

MeCN		
Species	E (Eh)	chem. pot. (kJ mol^{-1})
**1**	-2799.335134	953.70
**2**	-3028.411012	1088.57
**3**	-1877.642178	917.63
**4**	-2106.719895	1062.45
HCl	-460.823057	-36.61
**HL1**	-632.741959	481.40
**HL2**	-747.281867	553.65
Re**L1**	-2166.576003	410.02
Re**L2**	-2281.114401	482.84

$CHCl_3$		
Species	E (Eh)	chem. pot. (kJ mol^{-1})
**1**	-2799.319839	956.33
**2**	-3028.391208	1088.40
**3**	-1877.634031	918.39
**4**	-2106.709361	1063.44
HCl	-460.821716	-36.52
**HL1**	-632.721883	482.32
**HL2**	-747.263112	554.46
Re**L1**	-2166.548848	410.03
Re**L2**	-2281.084903	482.62

DCM		
Species	E (Eh)	chem. pot. (kJ mol^{-1})
**1**	-2799.327227	955.21
**2**	-3028.400676	1088.22
**3**	-1877.637967	918.06
**4**	-2106.714475	1062.73
HCl	-460.822382	-36.57
**HL1**	-632.732029	481.84
**HL2**	-747.272586	553.98
Re**L1**	-2166.562290	410.05
Re**L2**	-2281.099473	482.70
Toluene		
Species	E (Eh)	chem. pot. (kJ mol^{-1})
**1**	-2799.307346	958.33
**2**	-3028.375752	1089.26
**3**	-1877.627157	919.09
**4**	-2106.700704	1064.20
HCl	-460.820507	-36.45
**HL1**	-632.702567	483.08
**HL2**	-747.244983	555.25
Re**L1**	-2166.524508	410.08
Re**L2**	-2281.058676	482.07

MeOH		
Species	E (Eh)	chem. pot. (kJ mol^{-1})
**1**	-2799.334724	953.79
**2**	-3028.410446	1087.59
**3**	-1877.641973	917.64
**4**	-2106.719619	1062.44
HCl	-460.823023	-36.61
**HL1**	-632.741471	481.42
**HL2**	-747.281411	553.67
Re**L1**	-2166.575311	410.00
Re**L2**	-2281.113652	482.83

EtOH		
Species	E (Eh)	chem. pot. (kJ mol^{-1})
**1**	-2799.333652	954.08
**2**	-3028.409097	1088.37
**3**	-1877.641408	917.72
**4**	-2106.718921	1062.50
HCl	-460.822936	-36.60
**HL1**	-632.740206	481.49
**HL2**	-747.280232	553.72
Re**L1**	-2166.573549	410.02
Re**L2**	-2281.111708	482.84

EtOAc		

Species	E (Eh)	chem. pot. (kJ mol^{-1})
**1**	-2799.322832	955.96
**2**	-3028.394954	1086.00
**3**	-1877.635622	918.30
**4**	-2106.711456	1063.12
HCl	-460.821992	-36.54
**HL1**	-632.726110	482.19
**HL2**	-747.267069	554.26
Re**L1**	-2166.554386	410.04
Re**L2**	-2281.090914	482.62

References

1 Rigaku Oxford Diffraction, *CrysAlisPro*, 2024.

2 Sheldrick G. M., *Acta Cryst. C*, 2015, 3–8.

3 Sheldrick G. M., *Acta Cryst. A*, 2015, **71**, 3–8.

4 Dolomanov O. V., Bourhis L. J., Gildea R. J., Howard J. A. K. and Puschmann H., *J. Appl. Cryst.*, 2009, **42**, 339–341.

5 Treutler O. and Ahlrichs R., *J. Chem. Phys.*, 1995, **102**, 346.

6 Arnim M. and Ahlrichs R., *J. Comput. Chem.*, 1998, **19**, 1746–1757.

7 *TURBOMOLE V7.9 2024*, 2024.

8 Ahlrichs R., Bär M., Häser M., Horn H. and Kölmel C., *Chem. Phys. Lett.*, 1989, **162**, 165–169.

9 Perdew J. P., Burke K. and Ernzerhof M., *Phys. Rev. Lett.*, 1996, **77**, 3865–3868.

10 Grimme S., Antony J., Ehrlich S. and Krieg H., *J. Chem. Phys.*, 2010, **132**, 154104.

11 Grimme S., Ehrlich S. and Goerigk L., *J. Comput. Chem.*, 2011, **32**, 1456–1465.

12 Weigend F. and Baldes A., *J. Chem. Phys.*, 2010, **133**, 174102.

13 Klamt A. and Schüürmann G., *J. Chem. Soc., Perkin Trans. 2*, 1993, 799–805.

14 Figgen D., Peterson K. A., Dolg M. and Stoll H., *J. Chem. Phys.*, 2009, **130**, 164108.

15 Eichkorn K., Treutler O., Öhm H., Häser M. and Ahlrichs R., *Chem. Phys. Lett.*, 1995, **240**, 283–290.

16 Eichkorn K., Treutler O., Öhm H., Häser M. and Ahlrichs R., *Chem. Phys. Lett.*, 1995, **242**, 652–660.

17 Eichkorn K., Weigend F., Treutler O. and Ahlrichs R., *Theor. Chem. Acc.*, 1997, **97**, 119–124.

18 Weigend F., *Phys. Chem. Chem. Phys.*, 2006, **8**, 1057–1065.

19 Lin Y.-S., Li G.-D., Mao S.-P. and Chai J.-D., *J. Chem. Theory Comput.*, 2013, **9**, 263–272.

20 Becke A. D., *Phys. Rev. A*, 1988, **38**, 3098–3100.

21 Becke A. D., *J. Chem. Phys.*, 1993, **98**, 5648–5652.

22 Lee C., Yang W. and Parr R. G., *Phys. Rev. B*, 1988, **37**, 785–789.

23 Neese F., Wennmohs F., Hansen A. and Becker U., *Chem. Phys.*, 2009, **356**, 98–109.

24 Holzer C., *J. Chem. Phys*, 2020, **153**, 184115.

25 Plessow P. and Weigend F., *J. Comput. Chem.*, 2012, **33**, 810–816.
